# Chicken GSDME, a major pore-forming molecule responsible for RNA virus-induced pyroptosis in chicken

**DOI:** 10.1128/jvi.01588-24

**Published:** 2024-11-22

**Authors:** Zhi Chen, He Chang, Shujun Zhang, Hui Gao, Li Gao, Hong Cao, Xiaoqi Li, Yongqiang Wang, Shijun J. Zheng

**Affiliations:** 1 National Key Laboratory of Veterinary Public Health Security, Beijing, China; 2 Animal Epidemiology of the Ministry of Agriculture, Beijing, China; 3 College of Veterinary Medicine, China Agricultural University 34752 , Beijing, China; University of Michigan Medical School, Ann Arbor, Michigan, USA

**Keywords:** IBDV, pyroptosis, GSDME, caspase-3, caspase-7

## Abstract

Pyroptosis is an inflammatory type of programmed cell death that mainly depends on the formation of plasma membrane pores by Gasdermin D (GSDMD) in mammals. However, the genetic deficiency of GSDMD in chicken renders avian pyroptosis elusive. Here, we show that infection of DF-1 cells (a chicken cell line) with infectious bursal disease virus (IBDV) induced cell death associated with chicken GSDME (chGSDME) cleavage, and so did cells with other RNA virus (VSV, AIV, or NDV) infections, indicating a broad role of chGSDME in RNA virus-induced pyroptosis in chicken. Furthermore, infection of DF-1 cells by IBDV or treatment of cells with Poly(I:C) initiated MDA5-mediated signaling pathway, followed by the activation of chCaspase-3/7 cleaving chGSDME at a specific site _270_DAVD_273_. Moreover, knockdown or knockout of chGSDME expression in cells markedly reduced IBDV-induced pyroptosis and viral release. These results unravel the mechanisms of pyroptosis in chickens with RNA virus infection, providing important clues to uncover the role of GSDM proteins of different species in host response against pathogenic infection.

**IMPORTANCE:**

Pyroptosis is an inflammatory type of programmed cell death that mainly depends on the function of GSDMD in mammals and plays a crucial role in the pathogenesis of viral infection, whereas the mechanism of pyroptosis in chicken remains elusive. Herein, we show that IBDV and other RNA virus induced pyroptosis through the chMDA5-CASP8/9-CASP3/7-chGSDME pathway. The finding advances our understanding of GSDM proteins of different species in host response against pathogenic infection.

## INTRODUCTION

Pyroptosis is an inflammatory type of programmed cell death that mainly depends on the formation of plasma membrane pores by Gasdermin (GSDM) D in mammals (1–3). In addition to GSDMD, other members of GSDM family were also found to be involved in pyroptosis (4–8). Due to the genetic deficiency of GSDMD in chicken (9, 10), the mechanism of pyroptosis in chicken remains elusive. Elucidation of the mechanism underlying GSDM-mediated pyroptosis in chicken would much add to the understanding of the function of GSDM proteins and provide valuable guidance to the development of effective therapeutic agents for the control of infectious diseases.

Gasdermin family members are conserved proteins, including GSDMA, B, C, D, E, and DFNB59 (1, 11, 12). GSDM proteins contain pore-forming domain (PFD), repressor domain (RD), and a linker of divergence (11, 12). Among GSDM proteins, GSDMD is the best known pore-forming protein involved in pyroptosis (1–3), while other members of the Gasdermin family were also found to be involved in pyroptosis (4–8). Recent evidences reveal that GSDME, also called DFNA5, can be cleaved by caspase-3 or Granzyme B to induce pyroptosis (4, 5, 7). The cleavage of GSDME can switch caspase-3-mediated apoptosis induced by TNF or chemotherapeutic drugs to pyroptosis, offering new insights into cancer chemotherapy (4). In evolutionary terms, GSDME is the most ancient Gasdermin found in metazoans, and GSDME from different species with evolutionary distance to humans has been functionally characterized (13, 14). It was reported that GSDME from teleost fish and ducks is cleaved by caspase-1/3/7 or caspase-3/7 to induce pyroptotic cell death (15, 16). However, the role of GSDME-mediated pyroptosis in host response to pathogenic infections in chicken and the underlying mechanism remains to be elucidated.

Infectious bursal disease (IBD), originally called Gumboro disease, is an acute, highly contagious and immunosuppressive poultry disease caused by IBD virus (IBDV) (17). IBDV, a non-enveloped bi-segmented double-stranded (ds) RNA virus, belongs to the genus *Avibirnavirus* in the *Birnaviridae* family (18). The viral genomic segment A encodes VP2, VP3, VP4, and VP5, and Segment B encodes VP1, the viral RNA-dependent RNA polymerase (RdRp) (19, 20). It was well-established that IBDV infection caused cell death in bursa of Fabricius of chicken as well as in cell cultures (21–23). Among these five IBDV proteins, VP2 and VP5 are the major viral proteins involved in IBDV-induced cell death in the form of apoptosis (24–27), while other forms of cell death involved in IBDV infection process are still not very clear.

In this study, we found that infection of DF-1 cells (a chicken cell line) with IBDV induced pyroptosis associated with chGSDME cleavage, which was also true with other RNA virus (VSV, AIV, or NDV) infections. Importantly, infection of DF-1 cells by IBDV or treatment of cells with Poly(I:C) initiated MDA5-mediated signaling pathway, followed by the activation of chCaspase-3/7, which cleaved chGSDME at a specific site _270_DAVD_273_. Furthermore, knockdown or knockout of chGSDME expression in host cells markedly reduced IBDV-induced pyroptosis and viral release, indicating that chGSDME serves as a major membrane pore-forming molecule involved in pyroptosis caused by avian virus infection.

## RESULTS

### Infection of DF-1 cells by IBDV induces pyroptosis associated with chGSDME cleavage

**Fig 1:**
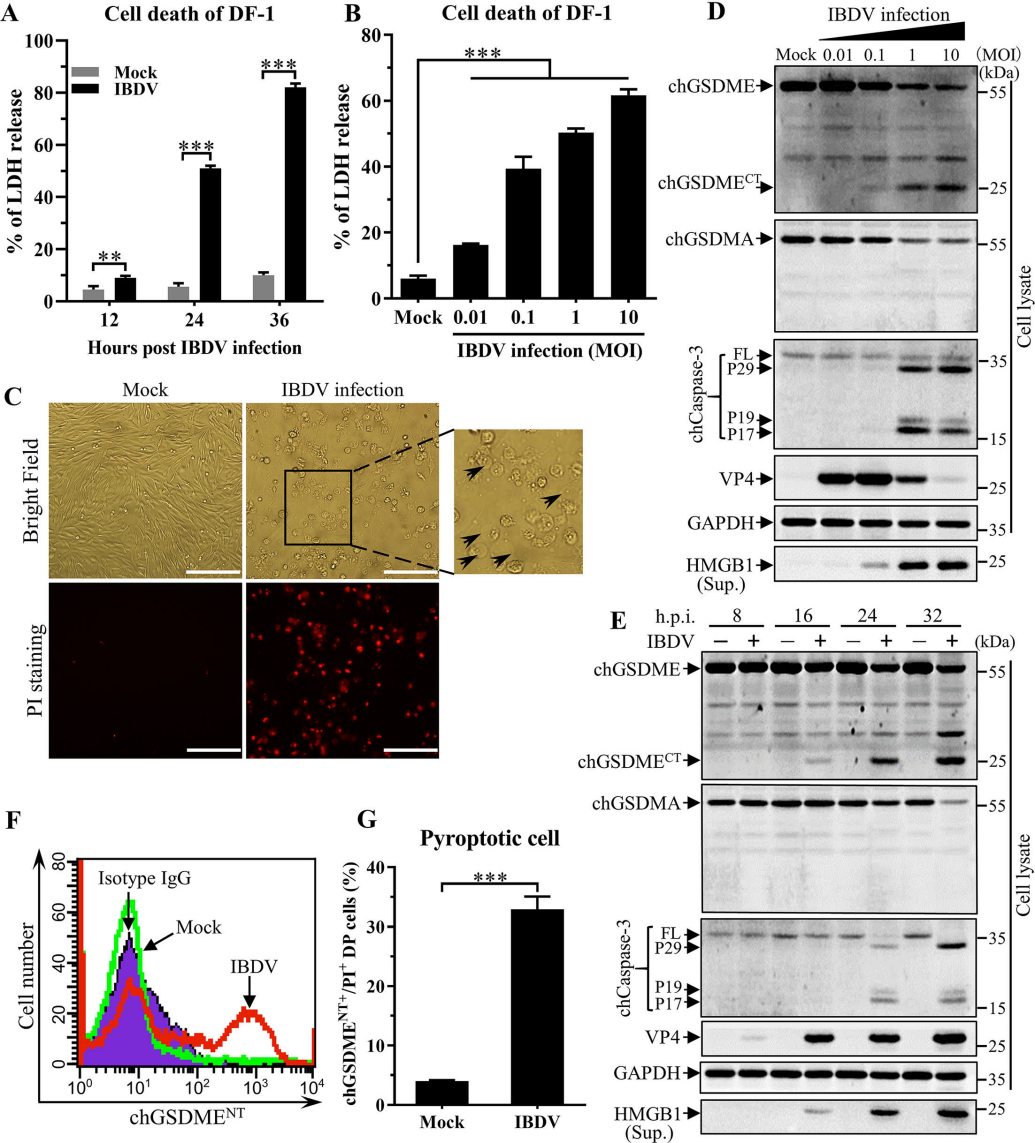
IBDV infection induces pyroptosis and cleavage of chicken Gasdermin E (chGSDME) in DF-1 cells. (A and B) Infection of DF-1 cells with IBDV induces LDH release. DF-1 cells were infected with IBDV at an MOI of 1 (**A**) or with different doses (at an MOI of 0.01, 0.1, 1, or 10) (**B**). LDH levels in the supernatant of cell culture were measured as described in Materials and Methods at indicated time points (12, 24, or 36 h) after IBDV infection (**A**) or at 24 h after IBDV infection with different doses (**B**). (**C**) Infection of DF-1 cells with IBDV induces pyroptotic cell death. DF-1 cells were infected with IBDV at an MOI of 1. Twenty-four hours after infection, cell morphology and PI staining-positive cells were observed under a fluorescence microscope, with arrows indicate pyroptotic cells. The scale bar in the picture represents 100  µm. (D and E) Infection of DF-1 cells with IBDV induced the cleavage of chGSDME. DF-1 cells were infected with IBDV with different doses (at an MOI of 0.01, 0.1, 1, or 10) (**D**) or at an MOI of 1 (**E**). At 24 h after IBDV infection with different doses (**D**) or at different time points (8, 16, 24, or 32 h) after IBDV infection (**E**), cell lysates and cell culture supernatants were examined with Western blot using anti-chGSDME, anti-chGSDMA, anti-Caspase-3, anti-VP4, anti-HMGB1, or anti-GAPDH antibodies; endogenous GAPDH expression was used as an internal control. (F and G) Examination of pyroptotic cells by flow cytometry. DF-1 cells were infected with IBDV at an MOI of 1. Twenty-four hours after infection, cells were harvested, stained with anti-chGSDME^NT^ McAb and PI, and analyzed with ﬂow cytometry. Histogram shows the cells with membrane-bound chGSDME^NT^ after IBDV infection (**F**), and the bar graph shows quantification of chGSDME^NT+^/PI^+^ DF-1 cells after IBDV infection (**G**). The data are representative of three independent experiments and presented as means ± SD. ***, *P*  <  0.001; **, *P*  <  0.01; *, *P*  <  0.05.

Previous studies by others and our laboratory showed that IBDV infection induced cell death in host cells *via* different pathways (23, 26, 28, 29). Thus, we speculated that IBDV infection might trigger pyroptosis. To test this hypothesis, we examined the release of lactate dehydrogenase (LDH), an indicator of the lytic cell death, in the cell culture with IBDV (*Lx* strain) infection. As a result, the contents of LDH in the supernatants of IBDV-infected cell culture dramatically increased compared with that of mock-infected controls (Fig. 1A and B), and IBDV infection induced LDH release in both time and dose-dependent manner, suggesting that IBDV infection induces lytic cell death in host cells. Interestingly, our data show that IBDV-infected cells exhibited morphological features of pyroptotic cell death, including cell swelling with large bubbles blowing from the plasma membrane and propidium iodide (PI)-staining positive (Fig. 1C), suggesting that IBDV-infected cells underwent pyroptosis.

As Gasdermins serve as crucial membrane pore-forming molecules in pyroptosis (30, 31), it was intriguing to assess Gasdermins involved in IBDV-induced pyroptosis. It was reported that chickens are genetically deficient of *Gsdmd* (9, 10), and chicken GSDMA (chGSDMA) and GSDME (chGSDME) are the currently known entire chicken Gasdermin family proteins with activities (14). chGSDMA and E (GenBank ID: NP_001026532.1 and NP_001006361.2) shared 30% and 51% amino acid identity with human GSDMA and E (GenBank ID: NP_835465.2 and XP_024302438.1). Thus, we set out to examine chGSDMA and chGSDME in cells with IBDV infection. As shown in Fig. 1D and E, the content of chGSDME in IBDV-infected cells markedly decreased compared with that of mock-infected controls in a dose- and time-dependent manner, whereas the content of C-terminal fragments of cleaved chGSDME in cells with IBDV infection remarkably increased and was associated with enhanced release of high-mobility group box 1 (HMGB1), an indicator of pyroptosis, indicating that IBDV infection caused pyroptosis (32, 33). Although chGSDMA expression in IBDV-infected cells was also downregulated, the cleaved chGSDMA was somehow undetectable. These data show that chGSDME is involved in IBDV-induced pyroptosis. We also observed that IBDV infection of CEF cells, the primary chicken cell lines, results in the cleavage of chGSDME and LDH release, suggesting that IBDV also induces pyroptosis of CEF cells (Fig. S1A and B).

Meanwhile, we examined the activation of caspase-3 in DF-1 cells with IBDV infection because caspase-3 is responsible for the activation of GSDME in mammals (4, 5). As a result, along with chGSDME cleavage in IBDV-infected cells, chicken caspase-3 (chCaspase-3) was also activated (Fig. 1D and E). These data suggest that chCaspase-3 might be involved in chGSDME-mediated pyroptosis in IBDV-infected cells.

As the formation of membrane pores of pyroptotic cells by N-terminal fragments of cleaved GSDM is a hallmark of pyroptosis, pyroptotic cells could be theoretically detected by flow cytometry *via* detecting N-terminal fragment of chGSDME on cell membrane using specific antibodies. Thus, we infected DF-1 cells with IBDV and examined pyroptotic cells by flow cytometry using monoclonal (McAb) antibodies against chGSDME^NT^ fragment (membrane-bound) and PI-staining. As a result, a considerable number of chGSDME^NT^-positive cells with IBDV infection were detected by flow cytometry assays (Fig. 1F), and the proportion of chGSDME^NT^/PI double-positive cells in IBDV-infected cells was significantly greater than that of mock-infected controls (Fig. 1G; Fig. S1C), indicating that IBDV-infected cells with membrane-bound chGSDME^NT^ were undergoing cell death. These data further consolidate the results that IBDV infection induces chGSDME-mediated pyroptosis in host cells.

### chGSDME^NT^ is required for pyroptosis in DF-1 cells

**Fig 2:**
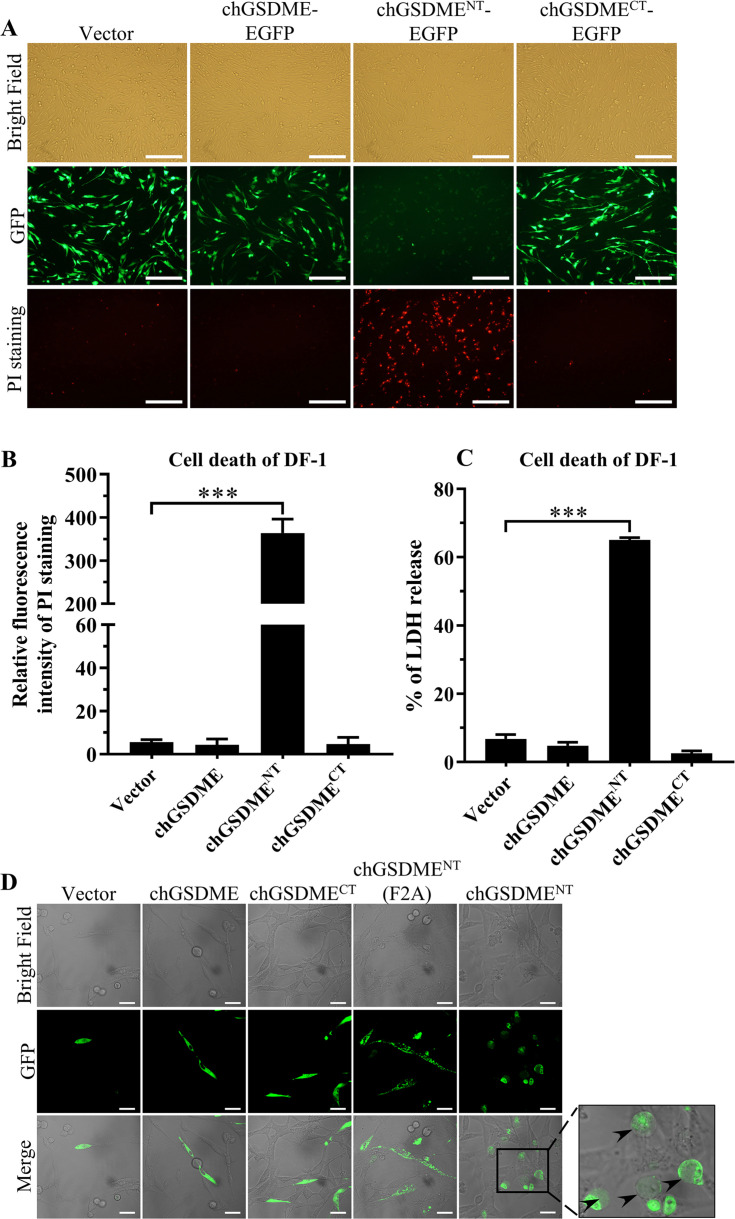
Expression of chGSDME^NT^ induces pyroptosis in DF-1 cells. DF-1 cells were transfected with plasmids encoding GFP, chGSDME-GFP, chGSDME^NT^-GFP, chGSDME^CT^-GFP, or mutant chGSDME^NT^(F2A)-GFP. Twenty-four hours after transfection, GFP-positive cells and PI staining-positive cells were observed under a fluorescence microscope (**A**); the scale bar in the picture represents 200  µm. Relative PI fluorescence intensity was detected by ImageJ (**B**). Release of LDH from the transfected cells was measured as described in Materials and Methods (**C**). Cell morphology was observed under a confocal laser scanning microscope (**D**); arrows indicate pyroptotic cells, and the scale bar in the picture represents 25  µm. The data are representative of three independent experiments and presented as means ± SD. ***, *P*  <  0.001; **, *P*  <  0.01; *, *P*  <  0.05.

As proteolytic cleavage of the Gasdermins in the linker region separates N- from C-terminal domains and the former subsequently form pores in cell membranes that lead to pyroptosis (30), we set out to investigate whether chGSDME was capable of inducing pyroptosis. First, we compared the sequence and conservative of chGSDME with human GSDME (HsGSDME). As shown in Fig. S2A and B, chGSDME shares 51% amino acid identity with HsGSDME and exhibits conserved structural features. Homology modeling analysis reveals that, similar to HsGSDME, chGSDME comprises a conserved NT domain (∼1–245 residues) and a CT domain (∼290–505 residues) connected with a flexible linker (246–289 residues), and chGSDME adopts a conserved three-dimensional structure similar to that of HsGSDME (Fig. S2B). Based on these results, we made pEGFP-chGSDME, chGSDME^NT^, and chGSDME^CT^ constructs, ectopically expressed these fusion proteins in DF-1 cells, and examined cell death in these cells. As a result, among these constructs, expression of GFP-chGSDME^NT^ in cells displayed severe cytotoxic effects as demonstrated by PI-staining positive cells and enhanced release of LDH from chGSDME^NT^-transfected cells (Fig. 2A through C). In contrast, expression of chGSDME or chGSDME^CT^ is mainly detected in the cytoplasm and induced very little LDH release. These data suggest that chGSDME^NT^ but not chGSDME or chGSDME^CT^ caused pyroptosis.

As we found that chGSDME^NT^ causes rapid changes in the membrane of pyroptotic cells (Fig. 2D), and that it is hard to directly observe the process of pyroptosis, we made a F2A mutant N-terminal domain construct to examine its membrane-targeting effect because the F2A mutant N-terminal domain was proved to retain the ability of targeting cell membrane but have reduced cytotoxicity (5). As shown in Fig. 2D, expression of chGSDME^NT^(F2A) could be observed on cell membranes, suggesting that the N-terminal fragment of chGSDME targets the plasma membrane to induce pyroptosis. These data further established that the N-terminal fragment of chGSDME is required for pyroptosis.

### Knockdown of chGSDME by RNAi inhibits IBDV-induced pyroptosis

**Fig 3:**
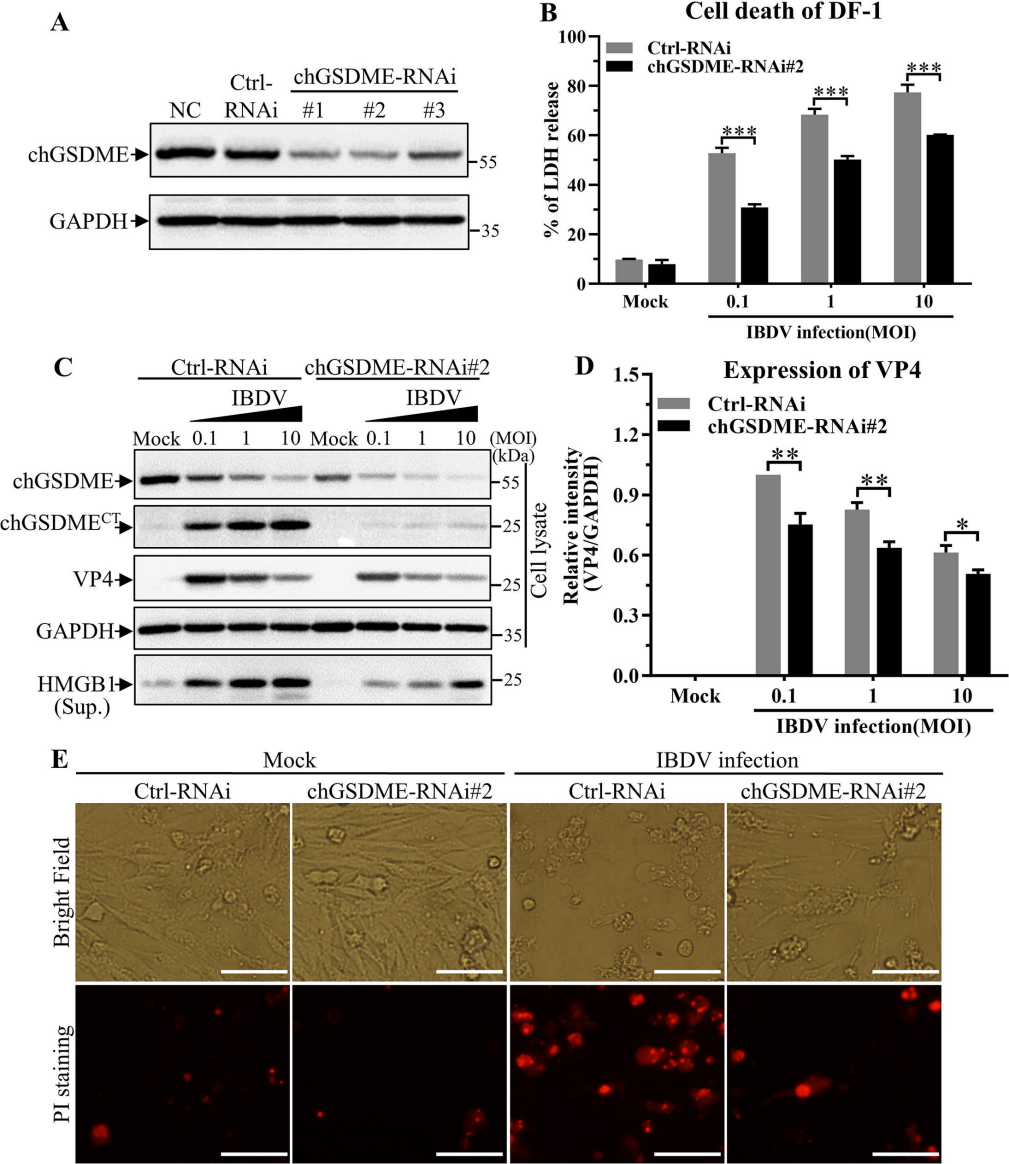
Knockdown of chGSDME by RNAi inhibits IBDV-induced pyroptosis. (**A**) Effects of chGSDME RNAi on the expression of endogenous chGSDME. DF-1 cells were double transfected with chGSDME-siRNA (RNAi#1 to 3) or siRNA control at a 24-h interval. Twenty-four hours after the second transfection, cell lysates were examined with Western blot using anti-chGSDME and anti-GAPDH antibodies; endogenous GAPDH expression was used as an internal control. (**B–E**) Knockdown of chGSDME by RNAi inhibits cell death and viral replication in IBDV-infected cells. DF-1 cells were double transfected with chGSDME-RNAi#2 as described above for panel A. Twenty-four hours after the second transfection, cells were infected with IBDV at different doses (at an MOI of 0.1, 1, or 10). Twenty-four hours post-infection, LDH levels in the supernatant of cell culture were measured as described in Materials and Methods (**B**). Cell lysates and cell culture supernatants were examined with Western blot using anti-chGSDME, anti-VP4, anti-HMGB1, and anti-GAPDH antibodies; endogenous GAPDH expression was used as an internal control (**C**). The band intensities for VP4 in panel C were quantitated by densitometry as shown in panel D. The relative levels of VP4 were calculated as the band density of VP4/that of GAPDH (**D**). Cell morphology and PI staining-positive cells were observed under a fluorescence microscope (**E**); the scale bar in the picture represents 50  µm. The data are representative of three independent experiments and presented as means ± SD. ***, *P*  < 0.001; **, *P*  <  0.01; *, *P*  < 0.05.

Since IBDV infection induced chGSDME cleavage and pyroptosis, we proposed that chGSDME might be involved in IBDV-induced pyroptosis. To test this hypothesis, we made three chGSDME RNAi constructs and found that chGSDME-RNAi#2 could effectively lower the cellular level of chGSDME without causing discernible changes in cell morphology (Fig. 3A). We then infected DF-1 cells receiving this siRNA or control siRNA with the IBDV *Lx* strain, and examined LDH levels in the supernatants of cell cultures. As shown in Fig. 3B, knockdown of chGSDME by RNAi markedly reduced LDH release from the cultured cells compared with that of controls (*P* < 0.001), indicating that knockdown of chGSDME expression mitigated IBDV-induced cell death. Meanwhile, we found that knockdown of chGSDME in DF-1 cells suppressed chGSDME cleavage and HMGB1 release as well as IBDV VP4 expression in IBDV- infected cells compared with that of controls (Fig. 3C and D). Likewise, PI staining-positive cells remarkably reduced in chGSDME RNAi transfected cells post-IBDV infection (Fig. 3E). These results clearly show that knockdown of chGSDME expression inhibits IBDV-induced pyroptosis associated with reduced viral replication.

### Knockout of chGSDME inhibits IBDV-induced pyroptosis and restricts viral growth

**Fig 4:**
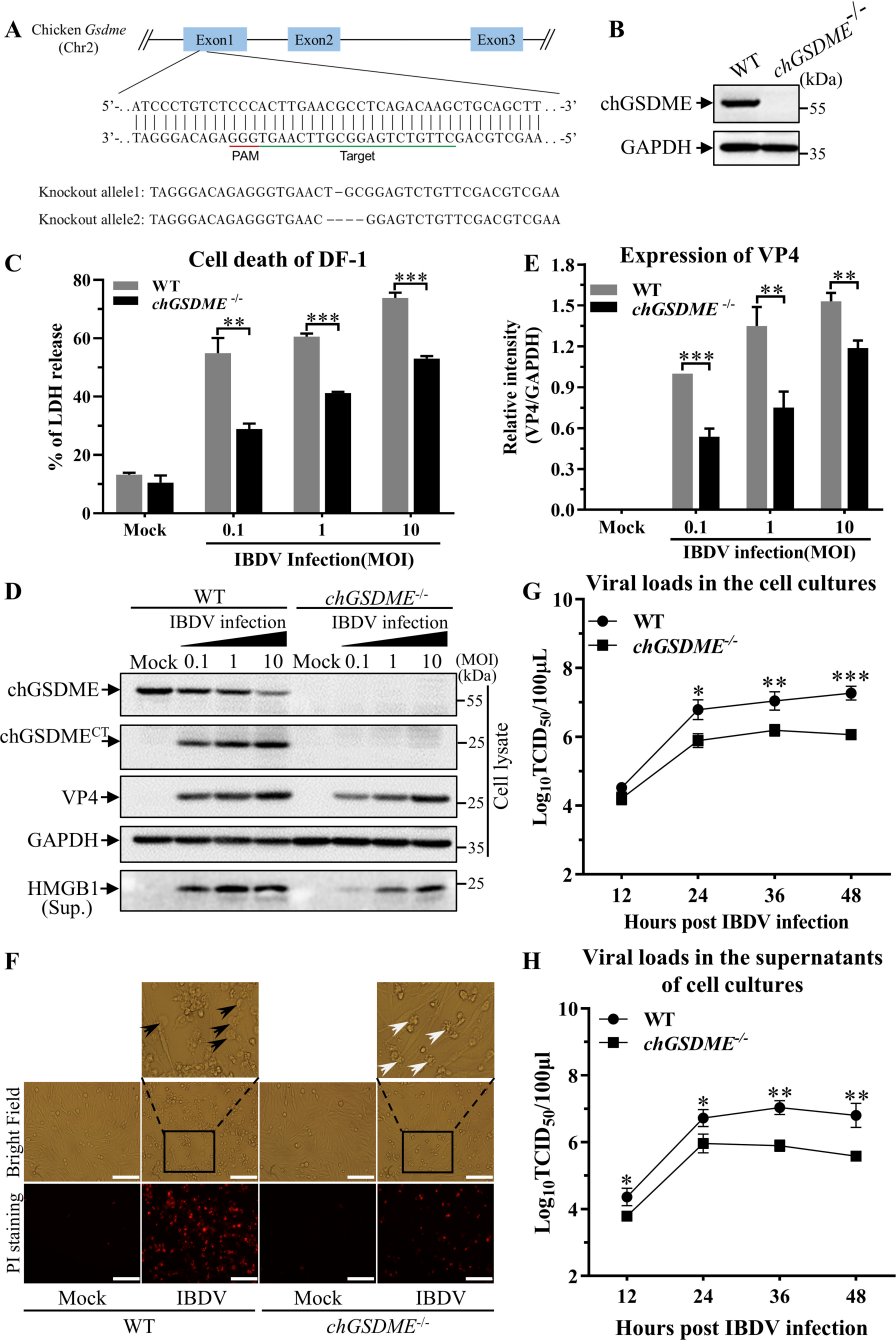
Knockout of chGSDME inhibits IBDV-induced pyroptosis and restricts viral release. (A and B) Generation of *chGsdme*^−/−^ DF-1 cells by CRISPR/Cas9-mediated genome editing. The panel A shows a related portion of the *chGsdme* genomic components; the target sites of *chGsdme* are underlined in green (**A**). Expression of chGSDME in WT and *chGsdme*^−/−^ cells was examined by Western blot using McAb against chGSDME (**B**). (**C–F**) Knockout of chGSDME inhibits cell death and VP4 expression in IBDV-infected cells. WT and *chGsdme*^−/−^ DF-1 cells were infected with IBDV at different doses (at an MOI of 0.1, 1, or 10). Twenty-four hours post-infection, LDH levels in the supernatant of cell culture were measured as described in Materials and Methods (**C**); cell lysates and cell culture supernatants were examined with Western blot using anti-chGSDME, anti-VP4, anti-HMGB1, and anti-GAPDH antibodies; endogenous GAPDH expression was used as an internal control (**D**). The band intensities for VP4 in panel D were quantitated by densitometry as shown in panel E. The relative levels of VP4 were calculated as the band density of VP4/that of GAPDH (**E**). Cell morphology and PI staining-positive cells were observed under a fluorescence microscope (**F**); black arrows indicate pyroptotic cells, and white arrows indicate apoptotic cells. The scale bar in the picture represents 100  µm. (G and H) Knockout of chGSDME restricts IBDV growth and viral release. *chGsdme*^−/−^ DF-1 cells and WT controls were infected with IBDV at an MOI of 0.5. At different time points (12, 24, 36, and 48 h) after IBDV infection, the viral loads in the cell cultures (**G**) and the supernatants (**H**) were determined by TCID_50_ assays using 96-well plates. The data are representative of three independent experiments and presented as means ± SD. ***, *P*  < 0.001; **, *P*  < 0.01; *, *P*  < 0.05.

The facts that knockdown of chGSDME expression in cells inhibits IBDV-induced pyroptosis and viral replication prompted us to further determine the role of chGSDME in pyroptosis and viral replication in IBDV-infected cells. We generated chGSDME-deficient DF-1 cell line by CRISPR-Cas9 system as demonstrated by Western blot analysis (Fig. 4A and B), infected the cells with IBDV, and examined LDH release and VP4 expression. As a result, deficiency of chGSDME in DF-1 cells markedly reduced LDH and HMGB1 releases compared with that of WT controls post-IBDV infection (Fig. 4C and D), and VP4 expression also decreased in chGSDME-deficient cells with IBDV infection compared with that of WT controls (Fig. 4D and E), suggesting that chGSDME was involved in pyroptosis and reduced viral replication. Furthermore, IBDV-induced cell death was remarkably reduced in cells deficient of chGSDME as examined by PI-staining assay (Fig. 4F), indicating that a large portion of cell death could be attributed to chGSDME-mediated pyroptosis. Moreover, deficiency of chGSDME in cells with IBDV infection significantly reduced viral growth as compared to that of WT controls as examined by TCID_50_ assays (Fig. 4G and H). These data further demonstrate that chGSDME is required for IBDV-induced pyroptosis and facilitates viral replication.

### IBDV-induced pyroptosis associated with chGSDME cleavage relies on caspase activities

**Fig 5:**
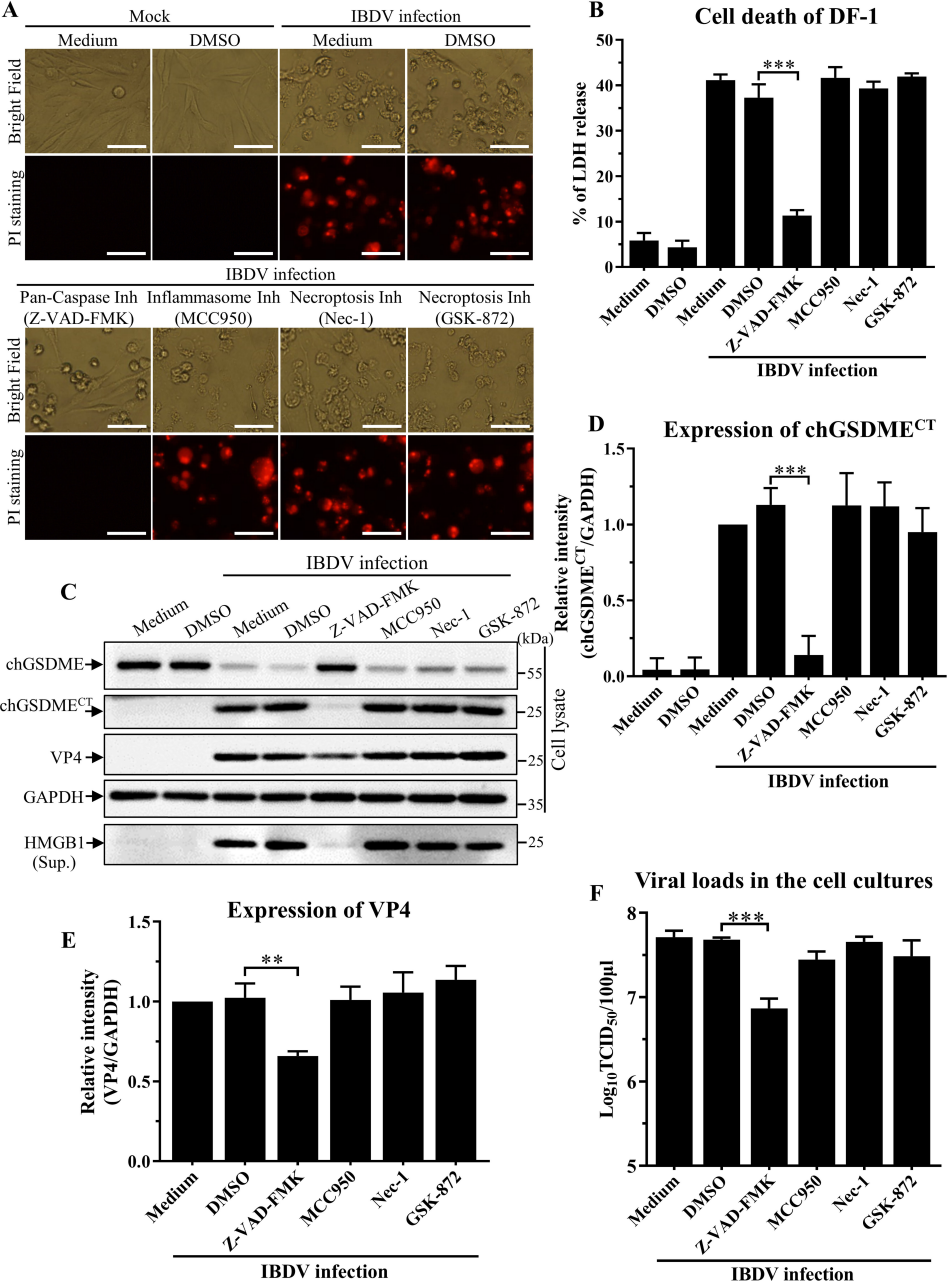
Pan-caspase inhibitor markedly alleviates IBDV-induced cell death associated with inhibition of chGSDME cleavage. (A and B) Pan-caspase inhibitor inhibits IBDV-induced cell death. DF-1 cells were infected with IBDV at an MOI of 1, followed by incubation with either 20 µM Z-VAD-FMK (pan-caspase inhibitor), 10 µM MCC950 (inflammasome inhibitor), 20 µM Nec-1 (necroptosis inhibitor), 5 µM GSK-872 (necroptosis inhibitor), or DMSO as control. Twenty-four hours post-infection, cell morphology and PI staining-positive cells were observed under a fluorescence microscope (**A**); the scale bar in the picture represents 50  µm. LDH levels in the supernatant of cell culture were measured as described in Materials and Methods (**B**). (**C–E**) Pan-caspase inhibitor inhibits IBDV-induced chGSDME cleavage and VP4 expression. DF-1 cells were treated as described above. Cell lysates and cell culture supernatants were examined with Western blot using anti-chGSDME, anti-VP4, anti-HMGB1, and anti-GAPDH antibodies; endogenous GAPDH expression was used as an internal control (**C**). The band intensities for chGSDME^CT^ or VP4 in panel C were quantitated by densitometry as shown in panel D or E. The relative levels of chGSDME^CT^ or VP4 were calculated as the band density of chGSDME^CT^ or VP4/that of GAPDH (D and E). (**F**) Pan-caspase inhibitor restricts IBDV growth. DF-1 cells were treated as described above. Twenty-four hours post-infection, the viral loads in the cell cultures were determined by TCID_50_ assays by using 96-well plates. The data are representative of three independent experiments and presented as means ± SD. ***, *P*  < 0.001; **, *P*  < 0.01; *, *P*  < 0.05.

Since our data show that chGSDME was required for IBDV-induced pyroptosis, we attempted to dissect the pathways for IBDV-induced pyroptosis associated with chGSDME cleavage. We treated IBDV-infected DF-1 cells with pan-caspase inhibitor Z-VAD-FMK, inflammasome inhibitor MCC950 or necroptosis inhibitors Nec-1 and GSK-872, examined the lytic cell death by measurement of LDH and HMGB1 release as well as PI-staining cells, and detected chGSDME cleavage by Western blot. As shown in Fig. 5A through D, IBDV-induced cell death, LDH and HMGB1 release, and chGSDME cleavage were significantly inhibited by Z-VAD-FMK (pan-caspase inhibitor) rather than by MCC950 (inflammasome inhibitor) or by Nec-1 and GSK-872 (necroptosis inhibitors). Additionally, Z-VAD-FMK also markedly reduced VP4 expression IBDV replication (Fig. 5E and F). These data indicate that IBDV-induced pyroptosis is highly dependent on caspase activities.

**Fig 6:**
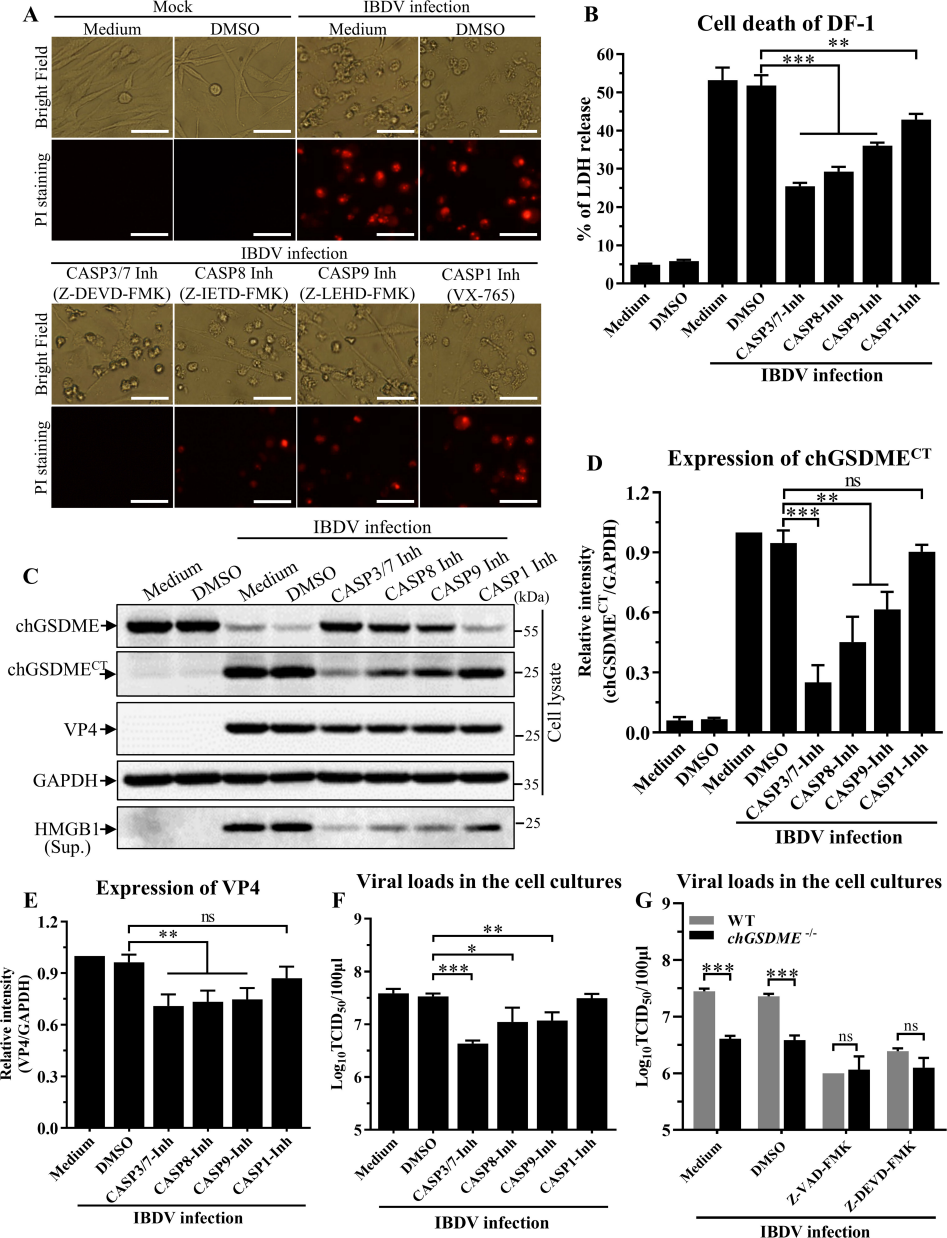
Inhibition of caspase activities by inhibitors suppressed IBDV-induced cell death and chGSDME cleavage. (A and B) Caspase inhibitors inhibited IBDV-induced cell death. DF-1 cells were infected with IBDV at an MOI of 1, followed by incubation with either 20 µM Z-DEVD-FMK (caspase-3/7 inhibitor), 20 µM Z-IETD-FMK (caspase-8 inhibitor), 20 µM Z-LEHD-FMK (caspase-9 inhibitor), 20 µM VX-765 (caspase-1 inhibitor), or DMSO as control. Twenty-four hours post-infection, cell morphology and PI staining-positive cells were examined under a fluorescence microscope (**A**), and LDH levels in the supernatant of cell culture were measured with LDH release assays (**B**). The scale bar in panel A represents 50  µm. (**C–E**) Caspase inhibitors inhibit IBDV-induced chGSDME cleavage and VP4 expression. DF-1 cells were treated as described above. Cell lysates and cell culture supernatants were examined with Western blot using anti-chGSDME, anti-VP4, anti-HMGB1, and anti-GAPDH antibodies, respectively. Endogenous GAPDH expression was used as an internal control. The band intensities for chGSDME^CT^ or VP4 in panel C were quantitated by densitometry as shown in panel D or E. The relative levels of chGSDME^CT^ or VP4 were calculated as the band density of chGSDME^CT^ or VP4/that of GAPDH (D and E). (**F**) Caspase inhibitors restrict IBDV growth. DF-1 cells were treated as described above. Twenty-four hours post-infection, the viral loads in the cell cultures were determined by TCID_50_ assays using 96-well plates. (**G**) Effects of caspase inhibitors on IBDV replication in WT and *chGsdme*^−/−^ DF-1 cells. WT and *chGsdme*^−/−^ DF-1 cells were infected with IBDV and treated with inhibitors as described above. Twenty-four hours post-infection, the viral loads in the cell cultures were determined by TCID_50_ assays using 96-well plates. The data are representative of three independent experiments and presented as means ± SD. ***, *P*  < 0.001; **, *P*  < 0.01; *, *P*  < 0.05; ns, not statistically significant.

To further determine which caspase was responsible for IBDV-induced chGSDME cleavage and pyroptosis, we infected DF-1 cells with IBDV and continued the cell culture in the presence of Z-DEVD-FMK (caspase-3/7 inhibitor), Z-IETD-FMK (caspase-8 inhibitor), Z-LEHD-FMK (caspase-9 inhibitor), or VX-765 (caspase-1 inhibitor). As a result, all four inhibitors were able to inhibit IBDV-induced pyroptosis as determined by PI staining of cells (Fig. 6A) and releases of LDH (Fig. 6B) and HMGB1 (Fig. 6C), but all inhibitors except for VX-765 (caspase-1 inhibitor) suppressed chGSDME cleavage as examined by Western blot analysis (Fig. 6C and D), but their inhibitory effects varied, with Z-DEVD-FMK (the caspase-3/7 inhibitor) achieving the greatest inhibitory effect. Additionally, VP4 expression IBDV replication was also reduced by apoptotic caspase inhibitors (Fig. 6E and F). These data indicate that IBDV-induced pyroptosis and chGSDME cleavage are highly dependent on caspase-3/7 activities.

We further examined the effect of caspase inhibitors on IBDV replication in WT and *chGsdme*^−/−^ DF-1 cells. Consistent with previous results (Fig. 4G and H, 5E and F, and 6E and F), knockout of chGSDME or the addition of caspase inhibitors restricts IBDV growth (Fig. 6G). However, knockout of chGSDME did not further reduce viral replication following the addition of pan-caspase inhibitor or CASP3/7 inhibitor, indicating that chCASP3/7 acts upstream of chGSDME to promote IBDV replication (Fig. 6G).

### chGSDME is a substrate directly cleaved by chCASP3 and 7

**Fig 7:**
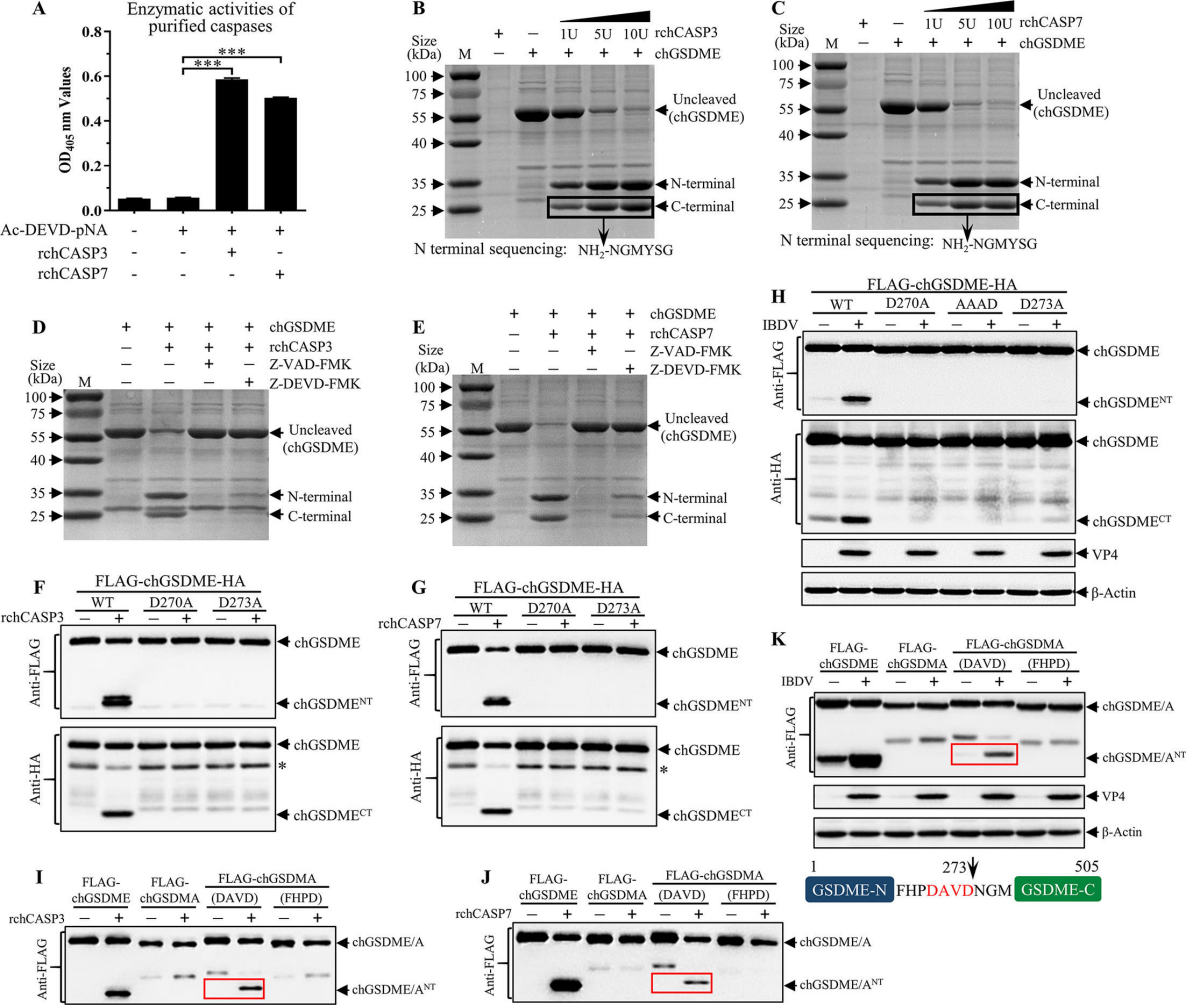
chCASP3 and chCASP7 cleave chGSDME at the _270_DAVD_273_ site. (**A**) Purified rchCASP3/7 exhibits enzymatic activities. The enzymatic activities of rchCASP3/7 were determined by measuring OD_405_ nm values after incubation with Ac-DEVD-pNA in caspase buffer at 37°C for 30 min. (B and C) *In vitro* cleavage of chGSDME by chCASP3/7; 5 µg of chGSDME was incubated with different units of chCASP3 (**B**) or chCASP7 (**C**) in caspase buffer at 37°C for 1 h. Cleavage of chGSDME was examined by Coomassie blue staining of the reaction samples separated on the SDS–PAGE gel. (D and E) The cleavage of chGSDME depends on enzymatic activity of chCASP3/7; 5 µg of chGSDME was incubated with 5U chCASP3 (**D**) or 5U chCASP7 (**E**) in the presence or absence of pan-caspase Inhibitor Z-VAD-FMK (2 µM) or caspase-3/7 inhibitor Z-DEVD-FMK (2 µM) at 37°C for 1 h. Cleavage of chGSDME was examined by Coomassie blue staining of the reaction samples separated on the SDS–PAGE gel. (F and G) Identification of the cleavage sites recognized by chCASP3/7 in chGSDME. HEK293T cells were transfected with plasmids encoding Flag-chGSDME-HA, Flag-chGSDME(D270A)-HA ,or Flag-chGSDME(D273A)-HA. Twenty-four hours after transfection, cell extracts were incubated with 10U chCASP3 (**F**) or 10U chCASP7 (**G**) at 37°C for 1 h, followed by immunoblotting with anti-Flag and anti-HA antibodies. (**H**) _270_DAVD_273_ is the key site for cleavage by IBDV-induced chGSDME. DF-1 cells were transfected with plasmids encoding WT chGSDME or mutant chGSDME. Twenty-four hours post-transfection, cells were infected with IBDV at an MOI of 5. Twenty-four hours post-infection, cell lysates were examined by Western blot with anti-Flag, anti-HA, anti-VP4, and anti-β-actin antibodies; endogenous β-actin expression was used as an internal control. (**I-K**) _270_DAVD_273_ is sufficient for the cleavage of chGSDME. DF-1 cells were transfected with plasmids encoding Flag-chGSDME, Flag-chGSDMA, Flag-chGSDMA(DAVD), or Flag-chGSDMA(FHPD). Twenty-four hours after transfection, cell extracts were incubated with 10U chCASP3 (**I**) or 10U chCASP7 (**J**) at 37°C for 1 h, and the cleavage of chGSDMA, chGSDME, or the mutants were examined by Western blot. Similarly, DF-1 cells were transfected as described above and infected with IBDV at an MOI of 5 for 24 h (**K**). The cleavage of chicken gasdermins was examined by Western blot. The data are representative of three independent experiments and presented as means ± SD. ***, *P*  < 0.001; **, *P*  < 0.01; *, *P*  < 0.05.

As caspase-3/7 inhibitor markedly inhibited chGSDME cleavage (Fig. 6C and D) and we noted that chGSDME contains residues 270–273 (_270_DAVD_273_) in the linker region (Fig. S2A), which was reported to act as putative caspase-3 and caspase-7 recognition motif as well as cutting site (34, 35), we set out to examine whether chGSDME is a substrate directly cleaved by chicken caspase-3 and caspase-7. We cloned chicken caspase-3 (chCASP3) and chicken caspase-7 (chCASP7) genes, expressed the active forms of recombinant chCASP-3/7 (rchCaspase-3/7) by prokaryotic expression system and purified rchCaspase-3/7 by affinity chromatography, and examined the enzymatic activities of rchCaspase-3/7 using colorimetric substrate Ac-DEVD-ρNA. As shown in Fig. 7A, rchCASP3 and rchCASP7 could catalyze Ac-DEVD-ρNA with sound enzymatic activity after incubation in caspase buffer at 37°C for 30 min. To determine the effect of rchCASP3 and rchCASP7 on chGSDME cleavage, we mixed 5 µg of chGSDME with different doses of rchCASP3 or rchCASP7 and examined the cleaved chGSDME with SDS-PAGE. As a result, both rchCASP3 and rchCASP7 could cleave chGSDME into N-terminal and C-terminal fragments in a dose-dependent manner (Fig. 7B and C). Furthermore, after sequencing C-terminal fragments by mass spectrometry analysis (S3A and B), we found that C-terminal fragments yielded from cleavage of chGSDME by both rchCASP3 and rchCASP7 began with the identical sequence _274_NGMYSG_279_, suggesting that the cleavage site of rchCASP3 and rchCASP7 in chGSDME is after Asp(D)^273^. Moreover, the cleavage of chGSDME by rchCASP3 or rchCASP7 could be abolished by pan-caspase Inhibitor Z-VAD-FMK or caspase-3/7 inhibitor Z-DEVD-FMK (Fig. 7D and E), indicating that the cleavage of chGSDME by rchCASP3 and rchCASP7 *in vitro* is specific.

To determine whether _270_DAVD_273_ is a bona fide recognition motif of chCaspase-3/7, we made mutant chGSDME constructs by replacing Asp (D) in _270_DAVD_273_ of chGSDME with Ala (A) separately, transfected HEK293T cells with the constructs carrying genes encoding mutant recombinant chGSDME having Flag-tag at N-terminus and HA-tag at C-terminus, and examined the cleavage of chGSDME in cell extracts treated with rchCaspase-3/7 with Western blot using anti-Flag and anti-HA antibodies. As a result, Flag-N-terminal fragment was readily detected with Western blot using anti-Flag antibodies, and so was HA-C-terminal fragment with anti-HA antibodies (Fig. 7F and G); however, the cleavage of chGSDME by rchCaspase-3/7 was completely abolished by D270A or D273A mutations, indicating that both D270 and D273 sites are crucial for the cleavage by rchCaspase-3/7. To further determine whether the _270_DAVD_273_ of chGSDME serves as the motif cleaved by endogenous chCaspase-3/7 in cells with IBDV infection, we transfected DF-1 cells with the constructs, and examined the cleavage of chGSDME in IBDV-infected DF-1 cells. Consistently, Flag-N-terminal fragment was readily detected with Western blot using anti-Flag antibodies, and so was HA-C-terminal fragment with anti-HA antibodies (Fig. 7H), whereas the cleavage of chGSDME could be completely abolished by D270A, D273A, or AAAD (triple alanine mutations of 270–272 residues) mutations in chGSDME in cells with IBDV infection (Fig. 7H), indicating that the presence of _270_DAV_272_ residues is required for the cleavage of chGSDME at Asp(D)^273^ by chCaspase-3/7. These results clearly show that chGSDME is directly cleaved by chCASP3 and chCASP7 at _270_DAVD_273_ in IBDV-infected cells.

To determine whether the _270_DAVD_273_ tetrapeptide motif is sufficient for the cleavage of chGSDME by chCaspase-3/7. We inserted this motif into chGSDMA to make mutant constructs by replacing _241_FASD_244_ of linker domain in chGSDMA with _270_DAVD_273_ in chGSDME or with _267_FHPD_270_ as control, transfected DF-1 cells with mutant chGSDMA (with DAVD or FHPD), WT chGSDMA, or chGSDME, and examined the cleavage of chGSDMA (with DAVD or FHPD) in cell extracts treated with rchCaspase-3/7 with Western blot assays using WT chGSDMA and chGSDME as negative and positive controls. As shown in Fig. 7I and J, WT chGSDMA was resistant to chCaspase-3/7 catalytic activity as demonstrated by Western blot, but the mutant chGSDMA carrying DAVD rather than FHPD linker, similar to WT chGSDME, became sensitive to chCaspase-3/7 cleavage and could be remarkably cleaved by chCaspase-3/7 to yield N-terminal fragment, suggesting that DAVD is a crucial motif that is required and sufficient for chGSDM cleavage by chCaspase-3/7. Consistently, N-terminal fragment of the mutant chGSDMA carrying DAVD in IBDV-infected cells could also be readily detected with Western blot, suggesting that the DAVD motif linker in chGSDMA makes the molecule sensitive to chCaspase-3/7 cleavage in cells with IBDV infection (Fig. 7K). Thus, the _270_DAVD_273_ tetrapeptide motif is required and sufficient for the cleavage of chGSDME by chCASP-3/7.

### The genomic dsRNA of IBDV induces chGSDME-mediated pyroptosis

**Fig 8:**
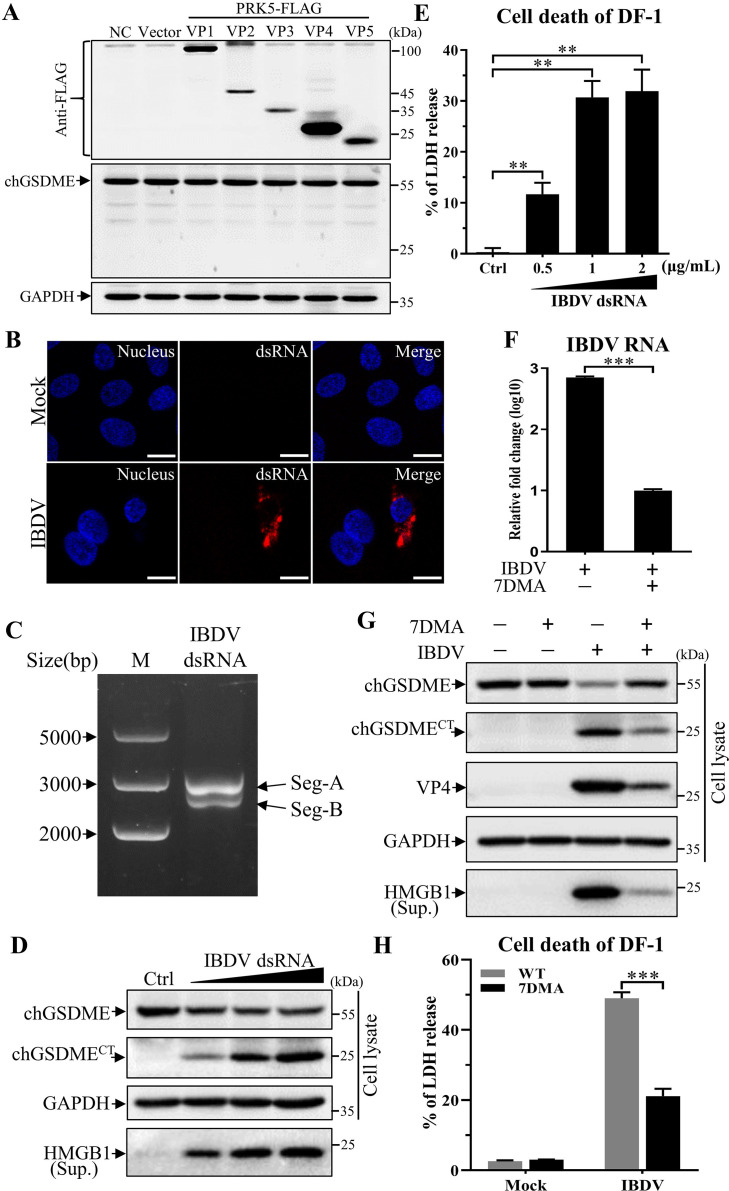
IBDV dsRNA activates the chGSDME-dependent pyroptosis. (**A**) IBDV viral protein do not induce chGSDME cleavage. DF-1 cells were transfected with PRK5 empty vectors or eukaryotic plasmids expressing viral protein-Flag fusions containing VP1, VP2, VP3, VP4, or VP5 of IBDV. Twenty-four hours post-transfection, cell lysates were examined with Western blot using anti-chGSDME, anti-Flag, and anti-GAPDH antibodies; endogenous GAPDH expression was used as an internal control. (**B**) Examination of dsRNAs in DF-1 cells infected with IBDV. DF-1 cells were stained with antibodies against dsRNA, and the cell nuclei were counterstained with DAPI at 16 h after IBDV infection. The cell samples were observed under a laser confocal scanning microscope. The scale bar in the picture represents 10  µm. (**C**) Examination of IBDV genomic dsRNA by electrophoresis; 0.2 µg dsRNA segments were separated in a 0.5% agarose gel, and the positions of two genomic segments are indicated. (D and E) IBDV dsRNA activates pyroptosis. DF-1 cells were transfected with IBDV dsRNA at different doses (0.5, 1, or 2 µg/mL) or transfection reagents as control. Twenty-four hours after transfection, chGSDME cleavage and HMGB1 release were examined with Western blot (**D**). LDH levels in the supernatant of cell culture were measured (**E**). (**F**) 7DMA inhibits IBDV dsRNA replication. DF-1 cells were infected with IBDV at an MOI of 1 and treated with 2.5 µM 7DMA at 4 h post-infection. At 16 h post-IBDV infection, total RNAs were extracted, and the expression levels of IBDV RNA were determined by qRT-PCR. (G and H) 7DMA inhibits IBDV-induced pyroptosis. DF-1 cells were infected with IBDV at an MOI of 1 and treated with 2.5 µM 7DMA at 4 h post-infection. Twenty-four hours after infection, chGSDME cleavage and HMGB1 release were examined with Western blot (**G**). LDH levels in the supernatant of cell culture were measured (**H**). The data are representative of three independent experiments and presented as means ± SD. ***, *P*  < 0.001; **, *P*  < 0.01; *, *P*  < 0.05.

As IBDV is a non-enveloped dsRNA virus, containing five viral proteins (VP1-5) and two segments of RNA genome, we proposed that one or more components of IBDV should be responsible for chGSDME cleavage in IBDV-infected cells. To test this hypothesis, we cloned the vp1, vp2, vp3, vp4, and vp5 genes from the IBDV *Lx* strain, made a Flag fusion for each of these proteins and expressed them in DF-1 cells by transfection. As shown in Fig. 8A, all five proteins were expressed well in this cell line, but none of these proteins showed any effect on chGSDME cleavage, indicating that IBDV viral proteins are not involved in IBDV-induced chGSDME cleavage.

We next examined the role of dsRNAs of IBDV genome in chGSDME cleavage. As shown in Fig. 8B, IBDV genomic dsRNAs were present in IBDV-infected cells, suggesting that the viral RNA might be recognized by RNA sensors and trigger host response. We successfully extracted dsRNAs from IBDV particles as shown in Fig. 8C, transfected DF-1 cells with the dsRNAs, and examined chGSDME cleavage in cells. As a result, transfection of cells with viral dsRNA markedly induced chGSDME cleavage, HMGB1 and LDH release in a dose-dependent manner (Fig. 8D and E), indicating viral dsRNA triggers chGSDME cleavage associated with pyroptosis. Importantly, inhibition of viral dsRNAs by 7-deaza-2′-C-methyladenosine (7DMA), a viral RdRp inhibitor (29, 36, 37), could significantly reduce the contents of viral dsRNAs in IBDV-infected cells (Fig. 8F), thereby decreasing IBDV-induced chGSDME cleavage and HMGB1 and LDH release (Fig. 8G and H). These data demonstrate that viral dsRNA, rather than other components of IBDV, induces chGSDME-mediated pyroptosis.

### Poly(I:C) and RNA virus induce chGSDME-mediated pyroptosis

**Fig 9:**
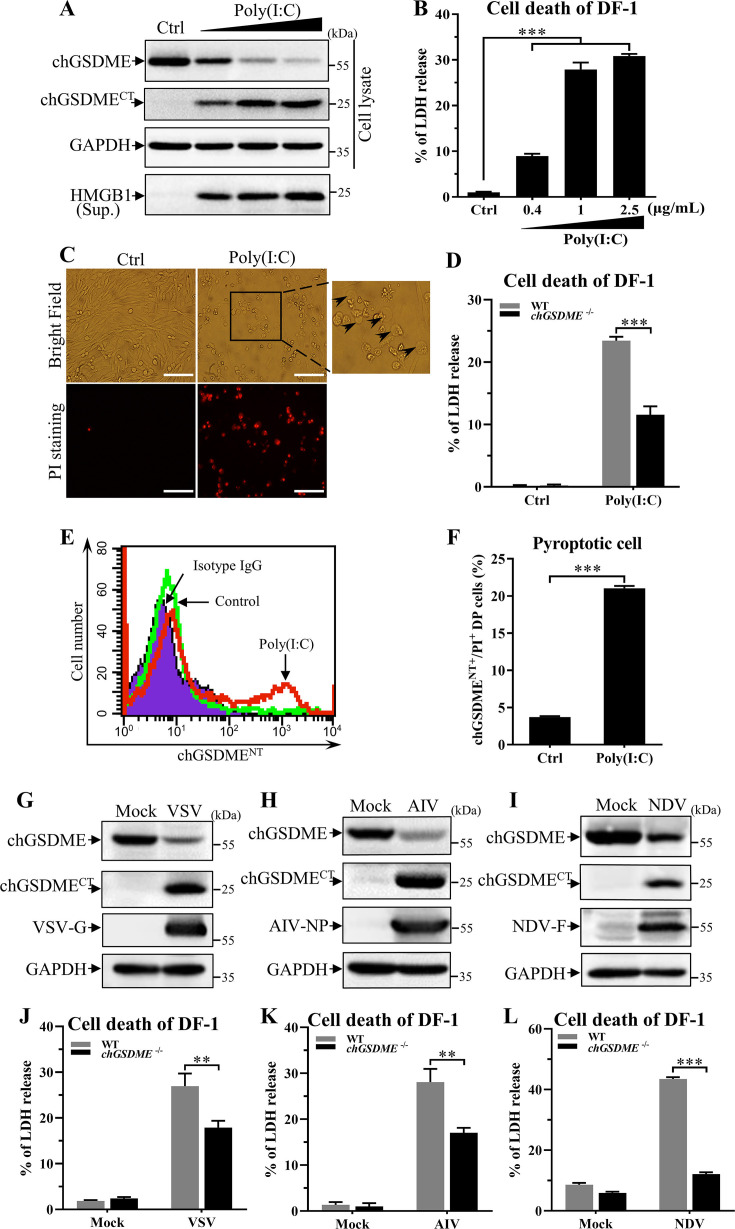
Poly(I:C) stimulation and RNA virus infection induce the chGSDME cleavage associated with cell death in host cells. (**A-C**) Poly(I:C) induces chGSDME cleavage associated with cell death. DF-1 cells were transfected with Poly(I:C) at different doses (0.4, 1, or 2.5 µg/mL) or transfection reagents as control. Twenty-four hours after Poly(I:C) stimulation, cell lysates and cell culture supernatants were examined with Western blot using anti-chGSDME, anti-HMGB1, and anti-GAPDH antibodies; endogenous GAPDH expression was used as an internal control (**A**). LDH levels in the supernatant of cell culture were measured as described in Materials and Methods (**B**). Cell morphology and PI staining-positive cells were observed under a fluorescence microscope (**C**); the scale bar in the picture represents 100  µm. (**D**) Knockout of chGSDME inhibits cell death in Poly(I:C)-stimulated cells. WT and *chGsdme*^−/−^ DF-1 cells were transfected with 1 µg/mL Poly(I:C) or transfection reagents as control. Twenty-four hours after Poly(I:C) stimulation, LDH levels in the supernatant of cell culture were measured. (**E-F**) Examination of pyroptotic cells by flow cytometry. DF-1 cells were transfected with 1 µg/mL Poly(I:C) or transfection reagents as control. Twenty-four hours after Poly(I:C) stimulation, cells were harvested, stained with anti-chGSDME^NT^ McAb and PI, and analyzed with ﬂow cytometry. Histogram shows the cells with membrane-bound chGSDME^NT^ after Poly(I:C) stimulation (**E**), and the bar graph shows quantification of chGSDME^NT+^/PI^+^ DF-1 cells after Poly(I:C) stimulation (**F**). (**G-I**) RNA virus (VSV, AIV, or NDV) induces cleavage of chGSDME. DF-1 cells were infected with VSV (MOI = 1), AIV (MOI = 1), or NDV (MOI = 0.1), respectively. At 18 (VSV) or 36 h (AIV and NDV) post-infection, cell lysates were examined with Western blot using anti-chGSDME, anti-VSV-G, anti-AIV-NP, anti-NDV-F, and anti-GAPDH antibodies; endogenous GAPDH expression was used as an internal control. (**J-L**) RNA virus (VSV, AIV, or NDV) infection induces chGSDME-dependent cell death. WT and *chGsdme*^−/−^ DF-1 cells were infected with VSV, AIV or NDV, as described above. LDH levels in the supernatant of cell culture were measured. The data are representative of three independent experiments and presented as means ± SD. ***, *P*  < 0.001; **, *P*  < 0.01; *, *P*  < 0.05.

To determine if chGSDME cleavage by viral dsRNA is IBDV-specific, we examined chGSDME activation in DF-1 cells stimulated with Poly(I:C), a synthetic dsRNA analog. As a result, transfection of cells with Poly(I:C) markedly induced chGSDME cleavage and enhanced HMGB1 and LDH release in a dose-dependent manner (Fig. 9A and B), and knockout of chGSDME in DF-1 cells significantly reduced LDH release (Fig. 9D). Consistently, the proportion of pyroptotic cells as examined by PI-staining, morphological observation, and flow cytometry markedly increased (Fig. 9C, E and F; Fig. S4). These results strengthen the findings that dsRNA is sufficient to cleave chGSDME and induce pyroptosis.

The facts that dsRNA induced chGSDME-mediated pyroptosis raised the possibility that chGSDME could also be activated by other RNA viruses. Thus, we infected DF-1 cells with Newcastle disease virus (NDV), Vesicular stomatitis virus (VSV), and Avian influenza virus (AIV) H9N2, and examined chGSDME cleavage and LDH release in virus-infected cells. Surprisingly, infection of DF-1 cells with these RNA viruses considerably induced chGSDME cleavage and LDH release, and the deficiency of chGSDME dampened RNA virus-induced cell death (Fig. 9G through L). These results suggest that chGSDME cleavage and pyroptosis induced by RNA virus were mainly attributed to the role of their RNA genome or viral RNA replication intermediates.

### chMDA5 is required for Poly(I:C)- or IBDV-induced chGSDME cleavage and pyroptosis

**Fig 10:**
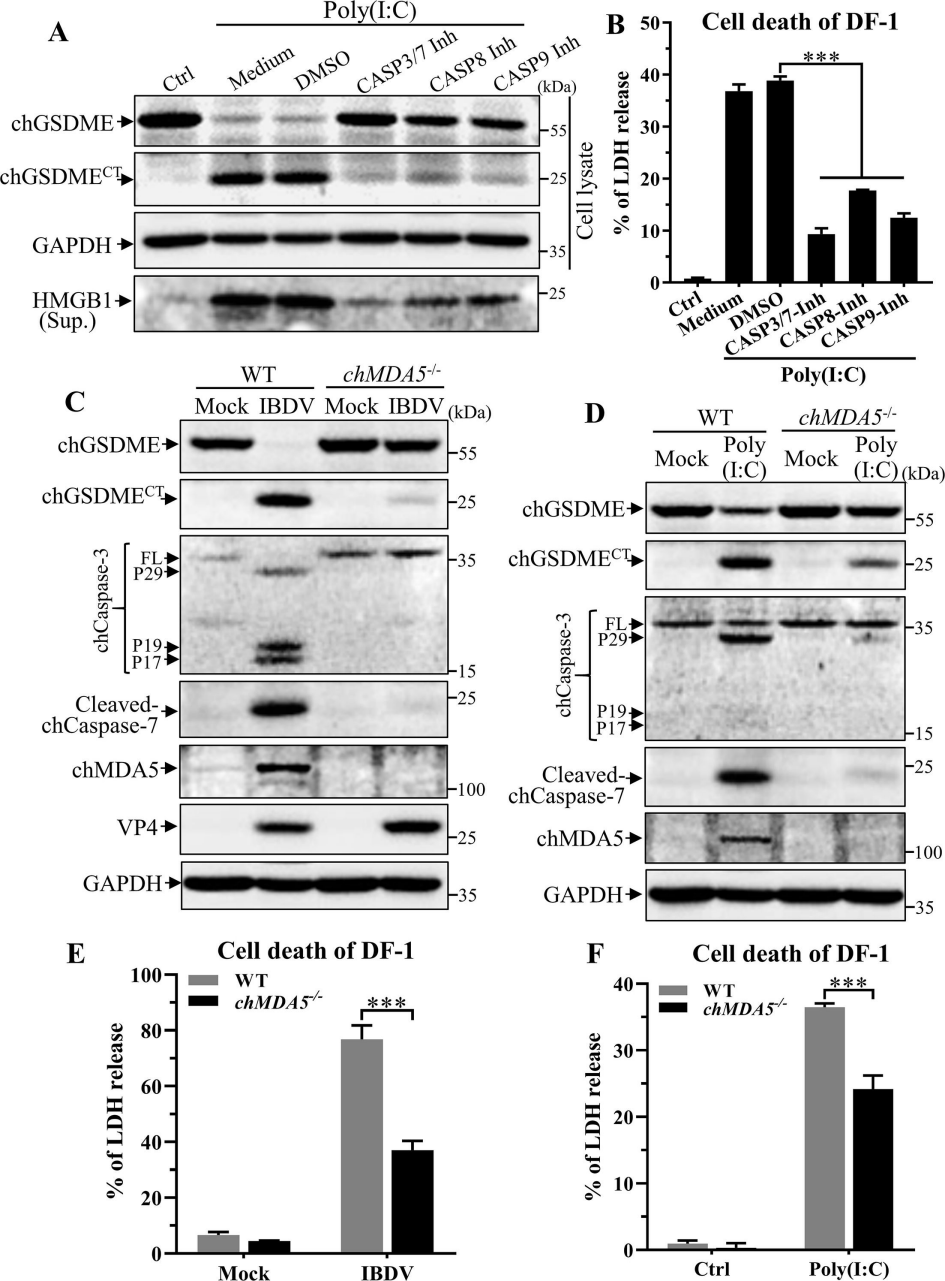
IBDV- or Poly(I:C)-induced cleavage of chGSDME and cell death were markedly reduced in *chMDA5*^−/−^ cells. (A and B) Caspase inhibitors inhibit Poly(I:C)-induced pyroptosis. DF-1 cells were transfected with 1 µg/mL Poly(I:C) or transfection reagents as control, followed by incubation with either 20 µM Z-DEVD-FMK, 20 µM Z-IETD-FMK, 20 µM Z-LEHD-FMK or DMSO as control. Twenty-four hours post-Poly(I:C) stimulation, cell lysates and cell culture supernatants were examined with Western blot using anti-chGSDME, anti-HMGB1, and anti-GAPDH antibodies; endogenous GAPDH expression was used as an internal control (**A**). LDH levels in the supernatant of cell culture were measured as described in Materials and Methods (**B**). (C and D) Knockout of chMDA5 inhibits IBDV- or Poly(I:C)-induced chCaspase-3/7 activation and chGSDME cleavage. *chMDA5*^−/−^ DF-1 cells and WT controls were infected with IBDV at an MOI of 10 (**C**) or transfected with 1 µg/mL Poly(I:C) (**D**). Twenty-four hours after infection or transfection, chCaspase-3/7 activation and chGSDME cleavage were examined with Western blot. (E and F) Knockout of chMDA5 inhibits IBDV- or Poly(I:C)-induced LDH release. *chMDA5*^−/−^ DF-1 cells and WT controls were infected with IBDV at an MOI of 10 (**E**) or transfected with 1 µg/mL Poly(I:C) (**F**). Twenty-four hours after infection or transfection, LDH levels in the supernatant of cell culture were measured. The data are representative of three independent experiments and presented as means ± SD. ***, *P*  < 0.001; **, *P*  < 0.01; *, *P*  < 0.05.

As Poly(I:C) is a synthetic dsRNA analog that can be used to mimic the PAMP of dsRNA virus to trigger immune response *via* engagement of retinoic acid inducible gene I (RIG-I)-like receptors (RLRs) (38, 39) and our data show that Poly(I:C) or IBDV-dsRNA induced chGSDME cleavage and pyroptosis, we set out to investigate whether chGSDME cleavage induced by Poly(I:C) or IBDV-dsRNA was dependent on caspase activation as IBDV did, and whether RLRs were involved in this process. We first examined the caspases involved in Poly(I:C)- or IBDV-dsRNA- induced chGSDME cleavage and LDH release using caspase inhibitors. As shown in Fig. 10A and B and Fig. S5A and B, treatment of cells by Poly(I:C) or IBDV-dsRNA markedly induced chGSDME cleavage and HMGB1 and LDH release, but this induction could be abolished by the CASP3/7, 8 and 9 inhibitors, which is consistent with the results observed in DF-1 cells infected with IBDV (Fig. 6A through D), and further established that the genomic dsRNA of IBDV is responsible for the virus-induced chGSDME cleavage and pyroptosis.

As RLRs are the cellular sensor for recognition of the genome of RNA virus in host cells, it would be intriguing to further determine the role of RLRs in Poly(I:C)- or IBDV-induced chGSDME activation and pyroptosis. Considering that RIG-I is genetically deficient in chickens (40, 41) and melanoma differentiation associated gene 5 (MDA5) is the key sensor of cytosolic viral RNA (42, 43), we examined the role of chicken MDA5 in chGSDME cleavage in WT and chMDA5-deficient (*chMDA5*^-/-^) DF-1 cells with IBDV infection or Poly(I:C) transfection. As shown in Fig. 10C, infection of DF-1 cells with IBDV markedly induced chGSDME cleavage and chCaspase-3/7 activation, but this induction was completely abolished in *chMDA5*^-/-^ DF-1 cells with IBDV infection. Similar results were also observed in *chMDA5*^-/-^ DF-1 cells with Poly(I:C) transfection (Fig. 10D). Consistently, LDH release in *chMDA5*^-/-^ cell culture was significantly reduced compared with that of WT cells post-IBDV infection or Poly(I:C) treatment (Fig. 10E and F). These results indicate that viral dsRNA initiates caspase activation *via* engagement of chMDA5, and as a consequence, the activated caspases cleave chGSDME, leading to pyroptosis.

**Fig 11:**
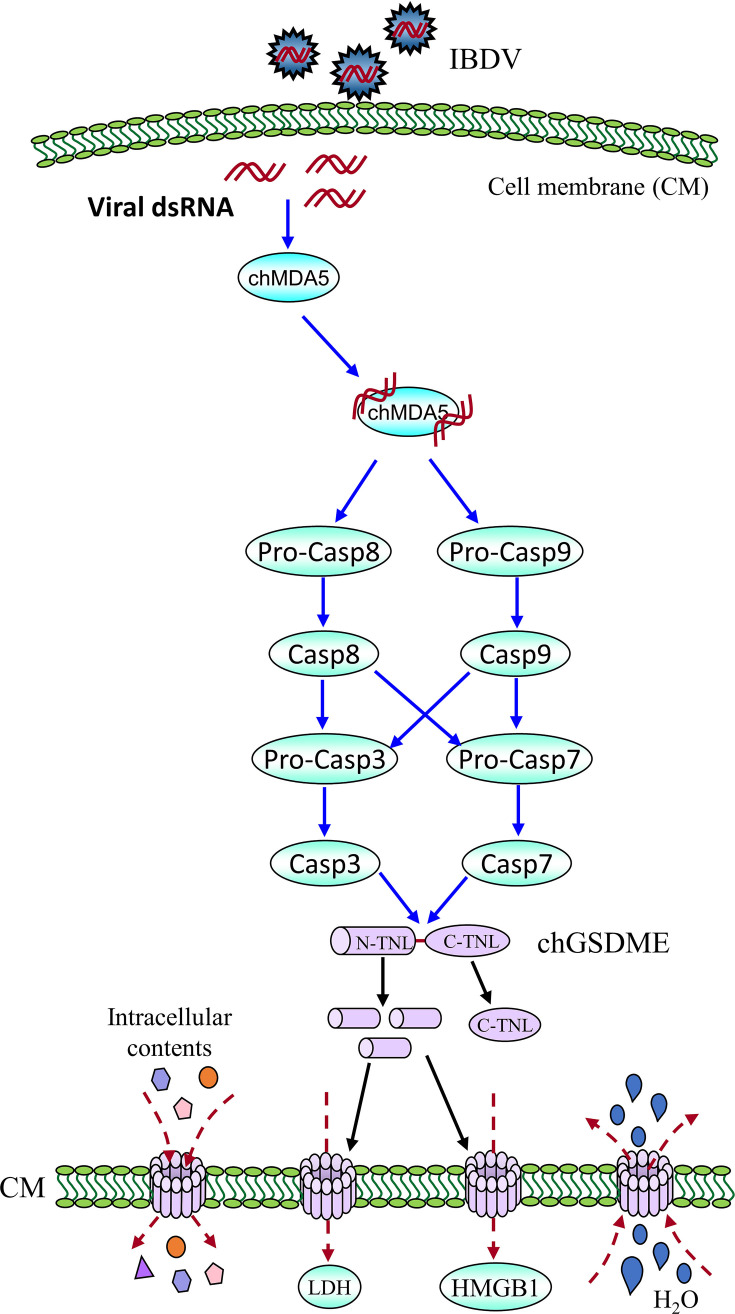
Graphic model of chGSDME-mediated pyroptosis in cells with IBDV infection. Recognition of IBDV dsRNA in host cells by cellular MDA5 initiates pro-caspase-8 and pro-caspase-9 cleavage, which subsequently activates caspase-3 and caspase-7. The activated caspase-3 and caspase-7 directly cleave chGSDME to separate the N-terminal domain (N-TNL) from the C-terminal domain (C-TNL), allowing the N-TNL to insert into cell membranes to form large oligomeric pores, which consequently lead to the influx of H_2_O and outflow of intracellular contents, including LDH, HMGB1, and so on, ultimately inducing cell death.

Taken together, our data show that, upon IBDV infection in chicken cells, viral genomic RNA was sensed by chMDA5 that activated caspase-8 and caspase-9, which subsequently activated caspase-3 and caspase-7, causing chGSDME cleavage and inducing LDH and HMGB1 release associated with cell death (Fig. 11).

## DISCUSSION

Pyroptosis, a programmed lytic cell death characterized by the formation of membrane pores by N-terminal fragment of cleaved Gasdermin and release of cytosolic contents including LDH, HMGB1 and other intracellular contents, is one of the most important components of inflammatory response as a consequence of pathogenic infection. GSDM cleavage associated with LDH release is the hallmark of pyroptosis (11, 44).

Gasdermin family members are conserved proteins, including GSDMA, B, C, D, E, and DFNB59 (1, 11, 12). GSDM proteins contain pore-forming domain (PFD), repressor domain (RD), and a linker of divergence (11, 12). Among GSDM proteins, GSDMD is the best known pore-forming protein involved in pyroptosis (1–3), other members of the Gasdermin family were also found to be involved in pyroptosis (4–8). Due to the genetic deficiency of GSDMD in chicken (9, 10), the mechanism of pyroptosis in chicken with pathogenic infections remains elusive. In the present study, we first found that IBDV infection induces GSDME cleavage and LDH and HMGB1 release as well as caspase activation (Fig. 1). Second, ectopic expression of N-terminal fragment of GSDME was sufficient to induce pyroptosis (Fig. 2). Third, knockdown or knockout of GSDME markedly inhibited IBDV-induced pyroptosis and restricted viral replication, indicating that chGSDME is required for IBDV-induced pyroptosis and facilitates viral replication (Fig. 3 and 4). Fourth, our data show that IBDV-induced chGSDME cleavage is dependent on caspase-3/7 activities, which directly cleaved chGSDME at _270_DAVD_273_, yielding N- and C-terminal fragments in host cells (Fig. 5 to 7). Importantly, we found that viral dsRNA is responsible for the chGSDME cleavage and pyroptosis in cells with IBDV infection, which is also true of VSV, NDV and AIV infections (Fig. 8 and 9). Finally, our data show that chMDA5 is required for viral genomic RNA-induced caspase activation, and the activated chCaspase-3/7 cleaved chGSDME, thereby leading to pyroptosis (Fig. 10). Therefore, our data reveal that engagement of viral RNA by chMDA5 ultimately induces activation of caspase-3/7, which cleaves chGSDME into N- and C-terminal fragments, and the N-terminal fragments form membrane pores in IBDV-infected cells, leading to pyroptosis (Fig. 11).

Infectious bursal disease (IBD), an immunosuppressive avian disease, poses severe threats to the poultry industry worldwide. The emergence of variant IBDV (vaIBDV) and very virulent IBDV (vvIBDV) strains caused severe economic losses to stakeholders in China as well as other countries (45–47). As the exact mechanism underlying IBDV-induced cell death is still not very clear, the development of effective vaccines encounters a bottleneck even though mutant virus could be rescued by reverse genetics. Thus, elucidation of the mechanism of IBDV infection is in urgent need. In this study, we explored the underlying mechanism of lytic cell death caused by IBDV infection and found that IBDV infection induced chGSDME-dependent pyroptosis in DF-1 cells, which promotes the release of viral particles and HMGB1. These results provide new insights into the pathogenesis of IBDV infection.

Pyroptosis is one of the most important defense strategies employed by the host to fight against viral infection (48). It was reported that GSDMD and GSDME were essential to limit IAV replication in NHBE cells (49), and inactivation of anti-apoptotic Bcl-2 family members induced by VSV infection promotes caspase-3/GSDME-dependent pyroptosis, and blocking this pathway enhanced viral replication in human skin organoids (50). Nevertheless, some viruses, particularly non-enveloped virus, require cell death to promote the release of viral particles from the infected cells. For example, norovirus induces mitochondrial dysfunction by encoding a MIKL-like protein, which leads to programmed cell death and viral egress (51). Our previous reports indicated that induction of apoptosis at a later stage of IBDV infection facilitated IBDV release (26–28). In the present study, knockout of chGSDME in DF-1 cells with IBDV infection inhibited pyroptosis and restricted viral growth, which might be attributed to the formation of membrane pores by N-terminal fragments from cleaved chGSDME that facilitate viral spread. Surprisingly, the proportion of pyroptotic cells could be readily determined by flow cytometry *via* detecting membrane-bound N-terminal fragments of chGSDME (chGSDME^NT^) using anti-chGSDME N-terminus antibodies (Fig. 1F). Importantly, the proportion of chGSDME^NT^/PI double-positive cells was around 33%, almost equal to that of chGSDME^NT^ positive cells (Fig. 1F and G; Fig. S1C), suggesting that the formation of membrane pores by chGSDME^NT^ was synchronous with the disruption of cell membranes, thereby cells undergo pyroptotic cell death. Likewise, around 21% of cells transfected with Poly(I:C) underwent pyroptosis as demonstrated by flow cytometry assay (Fig. 9D and E; Fig. S4). Thus, examination of pore-forming chGSDME^NT^ by flow cytometry using specific antibodies makes it possible to tell pyroptotic cells from others, thus determining the proportion of cells undergoing pyroptosis. Therefore, this method has an advantage over Western blot or IFA assay in detecting pyroptotic cells.

In addition to pyroptosis, apoptosis and necroptosis are the two main types of programmed cell death (PCD) caused by viral infection (52–54). Apoptosis is a non-inflammatory type of PCD that is initiated through either intrinsic or extrinsic pathway and eventually results in the activation of executioner caspase-3/7 (55). Necroptosis is an inflammatory form of PCD mediated by the serine/threonine kinase receptor interacting protein kinase 3 (RIPK3) and its downstream effector mixed-lineage kinase domain-like (MLKL) (56). It was previously thought that apoptosis and pyroptosis seem to have nothing in common, as their signaling pathways and morphology are completely different (57). Recently, however, GSDME was reported to be cleaved by caspase-3 upon apoptotic stimuli, which causes pyroptosis directly or induces secondary necrosis after apoptosis (4, 5). Similarly, GSDMD can also be directly processed by caspase-8 under certain conditions (58, 59). Thus, there is an extensive crosstalk between apoptosis and pyroptosis. Lines of evidence indicate that IBDV infection induced apoptosis (23, 28, 29). However, in this study, we found that around 33% of IBDV-infected cells underwent pyroptosis associated with chGSDME cleavage, LDH and HMGB1 release, morphological changes and PI-staining positive, and our data further show that caspase-3/7 directly cleaved chGSDME, triggering pyroptosis. This is the first time to provide solid evidence at a protein level that IBDV genomic dsRNA induced pyroptosis. However, how pyroptosis crosstalk with apoptosis and the exact underlying mechanism for the transition of apoptosis to pyroptosis are not clear. Further efforts will be necessary to fully elucidate the bidirectional crosstalk between apoptosis and pyroptosis upon IBDV infection.

A previous study indicated that IBDV persistently infects DT40 cells (60), which is an ALV-transformed bursal lymphoma cell line, suggesting that DT40 cells might not undergo pyroptosis. This could be attributed to the fact that a large proportion (>90%) of the total cell population remains uninfected (60), resulting in a lack of significant viral titer increase, which is necessary to produce viral genome dsRNA and activate pyroptosis. Additionally, GSDME expression varies across different cell types, which may influence whether a cell undergoes apoptosis or pyroptosis (4). When GSDME is highly expressed, caspase-3 can cleave GSDME to trigger pyroptosis, otherwise, it triggers apoptosis (61, 62). We examined the expressions of chGSDME in various chicken cell lines and found that the expression of chGSDME in DT40 cells was barely detectable as examined by Western blot assay using anti-chGSDME monoclonal antibody (data not shown), which might also be the reason why they do not undergo pyroptosis. Further investigation will be necessary to assess pyroptosis in additional chicken cell lines and in the bursa of Fabricius.

Human GSDMA or mouse GSDMA1 are recently found to be cleaved by cysteine protease SpeB of group A *Streptococcus* (GAS) and trigger keratinocyte pyroptosis (63, 64). Our data showed that chGSDMA expression was downregulated after IBDV infection, but no cleavage of chGSDMA was detected in IBDV-infected cells (Fig. 1D and E). The biological role of chGSDMA in IBDV-induced pyroptosis is still not clear. More efforts will be required to investigate the biological function of chGSDMA.

Pyroptosis is an inflammatory form of programmed cell death (PCD) that critically depends on the formation of plasma membrane pores by members of the Gasdermin family (65). Classical pyroptosis is executed by the generation of N-terminal fragmentation of Gasdermin D (GSDMD) *via* cleavage by inflammatory caspases, such as caspase-1 and caspase-4/5/11 (1–3). The cleavage of GSDMD at _273_LLSD_276_ in mouse or _272_FLTD_275_ in human liberates the N-terminal cytotoxic domain from the C-terminal autoinhibitory domain, and the released N-terminal fragments bind to phosphoinositides in the plasma membrane and oligomerize to form membrane pores, allowing the release of proinflammatory cytokines, such as the mature form of interleukin-1β (IL-1β) and IL-18 (30, 66). In the present study, our data show that chCaspase-3/7 could cleave chGSDME by recognition of the tetrapeptide motif _270_DAVD_273_ in the linker region. It is worth noting that the DAVD tetrapeptide motif is necessary and sufficient for the cleavage of chGSDME by caspase-3/7 because replacement of Asp (D) in _270_DAVD_273_ by Ala (A) totally abolished the caspase-3/7-induced cleavage of chGSDME, while insertion of DAVD motif into the linker domain of chGSDMA made mutant chGSDMA readily cleaved by caspase-3/7 (Fig. 7I through K). Considering that chicken Gasdermin family proteins contain only GSDMA and GSDME, we initially speculated that chGSDME might be cleaved by caspase-1. However, we did not find any evidence that caspase-1 was involved in chGSDME cleavage (Fig. 6), and instead, we found that chGSDME cleavage was mainly related to caspase-8, caspase-9, and caspase-3/7 using caspase inhibitors, and that chGSDME could be directly cleaved by caspase-3 and caspase-7 as demonstrated by *in vitro* assay (Fig. 7B through E). Therefore, it is very likely that engagement of chicken MDA5 by the genomic dsRNA of IBDV initiated activation of caspase-8 and caspase-9, which subsequently activated caspase-3/7 that cleaved chGSDME, leading to pyroptosis.

The viral RNA as the major pathogen-associated molecular pattern (PAMP) of RNA virus can be recognized by RLRs during viral infection of host cells (42). In the case of IBDV infection, it was reported that chMDA5 is the major cytoplasmic sensor for recognizing IBDV genomic dsRNA and initiates type I interferon (IFN) production (67). Our data show that IBDV dsRNA, rather than viral protein, induces chGSDME cleavage and pyroptosis, and that chMDA5 is essential for inducing chCaspase-3/7 activation and chGSDME cleavage upon IBDV infection, expanding our understanding of the function of chMDA5 beyond the induction of IFN expression.

Recently, several studies have shown that different PRRs activate pyroptosis by sensing viral genomic RNA or viral dsRNA formed during the course of viral replication (49, 62, 68). For instance, Semliki Forest virus (SFV) triggers NLRP1 inflammasome activation in keratinocytes by producing dsRNA (68), Influenza A virus (IAV) activates a PKR-caspase-8-caspase-3-GSDME signaling pathway in NHBE cells (49), and the genomic 5′ untranslated region (UTR) RNA of Zika virus (ZIKV) activates GSDME-dependent pyroptosis through the RIG-I–caspase-8–caspase-3 pathway (62). Consistent with these findings, our results also highlight the important role of viral RNA in inducing pyroptosis. In addition to IBDV, we tested other three RNA viruses (VSV, IAV, and NDV) and found that chGSDME cleavage and LDH release were also induced in DF-1 cells infected with VSV, IAV, and NDV (Fig. 9), suggesting that chGSDME-mediated pyroptosis may be a universal phenomenon as a consequence of RNA viral infection in chicken.

It was reported that MDA5 or RIG-I could induce caspase-3 activation in different ways (69–75). In melanoma cells, activation of RIG-I or MDA5 induced the expression of proapoptotic proteins, such as Noxa, Puma, Bim, and Bik, which activated the intrinsic apoptotic pathway (69). Furthermore, activation of RIG-I or MDA5 also increased the expression of tumor necrosis factor-related apoptosis-inducing ligand (TRAIL), which initiated extrinsic apoptotic pathway (70–72). In addition to promoting the expression of pro-apoptotic proteins, RIG-I was also reported to be involved in the activation of BAX by cytoplasm-to-mitochondria translocation of IRF-3, which causes cytochrome c release from the mitochondria (73, 74). Moreover, it was found that activation of MDA5 induced recruitment of caspase-8 to MAVS on mitochondria where caspase-8 is activated (75). In the present study, our data show that IBDV- or Poly(I:C)-induced chGSDME cleavage depended on the activation of caspase-8, caspase-9, caspase-3, and caspase-7 but not caspase-1, suggesting that viral RNA triggers the chMDA5–caspase–chGSDME pathway to induce pyroptosis.

In summary, our data show that infection of DF-l cells by IBDV induced chGSDME cleavage, LDH and HMGB1 release, and cell death. Engagement of chMDA5 by viral dsRNA or Poly(I:C) induced chGSDME cleavage, LDH and HMGB1 release associated with cell death. Importantly, our results show that caspase-8,caspase-9, caspase-3, and caspase-7 but not caspase-1 are involved in chGSDME cleavage, and that chGSDME could be directly cleaved by caspase-3 or caspase-7, suggesting that viral RNA triggers the chMDA5–caspase–chGSDME pathway to induce pyroptosis. Furthermore, we found that examination of pore-forming chGSDME^NT^ by flow cytometry using specific antibodies makes it possible to differentiate chGSDME-mediated pyroptotic cells from ones with other forms of cell death, and to determine the proportion of chGSDME^NT^/PI double-positive cells, which indicates that the formation of membrane pores by chGSDME^NT^ is synchronous with the disruption of cell membranes, exhibiting pyroptosis. Moreover, chGSDME cleavage and LDH release could be induced not only by IBDV but also by other RNA viruses, such as VSV, IAV, and NDV, indicating a crucial role of viral RNAs in pyroptosis in host cells. Our findings provide new insights into RNA virus-induced pyroptosis in nonmammalian species.

## MATERIALS AND METHODS

### Cells and virus

DF-1 (immortal chicken embryo fibroblasts) and HEK293T cells were obtained from ATCC. DF-1 and HEK293T cells were cultured in Dulbecco Modified Eagle Medium (DMEM, Macgene Biotechnology, China) supplemented with 10% fetal bovine serum (FBS, Gibco, USA) in a 5% CO_2_ incubator at 38°C. IBDV *Lx* strain was kindly provided by Dr. Jue Liu (Beijing Academy of Agriculture and Forestry, Beijing, China), NDV F48E9 strain by Dr. Jinhua Liu (China Agricultural University, Beijing, China), AIV-H9N2 A/chicken/Hebei/1/2017 strain by Dr. Yipeng Sun (China Agricultural University, Beijing, China), and VSV by Dr. Ruoqian Yan (Animal Disease Control Center of Henan Province, China).

### Reagents, chemicals and antibodies

Lipofectamine 3000 transfection reagent and RNAiMAX were purchased from Thermo Fisher Scientific (USA), the jetPRIME transfection reagent from Polyplus-transfection (France), and Poly(I:C) and PI (P4170) from Sigma-Aldrich (USA). Z-VAD-FMK (HY-16658B), Z-DEVD-FMK (HY-12466), Necrostatin-1 (HY-15760), GSK-872 (HY-101872), MCC950 (HY-12815A), and VX-765 (HY-13205) were purchased from MCE (USA), Z-IETD-FMK (B3232), and Z-LEHD-FMK (B3233) from APExBIO (USA). Mouse anti-VP4 antibody (EU0206), mouse anti-σB antibody (EU0210), mouse anti-chGSDM-A/E (EU0226, EU0228), and chGSDME^NT^ (EU0227) antibodies were purchased from SAE Biomed-tech (ZS) Company (China). Rabbit anti-VSV-G antibody (183497) was purchased from Abcam (United Kingdom), rabbit anti-Caspase-3 antibody (9662) and rabbit anti-HA antibody (3724) from CST (USA). Mouse anti-Flag antibody (F1804) was purchased from Sigma-Aldrich (USA), goat anti-Mouse IgG H, and L AF647 antibody (bs-0296G-AF647) from Bioss (China), mouse anti-GAPDH antibody (60004–1-Ig) from Proteintech (China), rabbit anti-GFP antibody (AE011), rabbit anti-cleaved caspase-7 (A23154), rabbit anti-HMGB1 antibody (A2553) and rabbit anti-β actin antibody (AC026) from ABclonal (China). Mouse anti-dsRNA antibody was kindly provided by Dr. Chen Peng (China Agricultural University, Beijing, China), and rabbit anti-NP antibody by Dr. Yipeng Sun (China Agricultural University, Beijing, China). Polyclonal antibodies against chicken MDA5 and NDV-F protein were prepared in our laboratory.

### Plasmid construction

PRK5-Flag-VP1, PRK5-Flag-VP2, PRK5-Flag-VP3, PRK5-Flag-VP4, and PRK5-Flag-VP5 were constructed previously by our laboratory (76, 77). Chicken *GSDME* (GenBank accession number: NM_001006361.2), *GSDMA* (GenBank accession number: NM_001031361.1), *Caspase-3* (GenBank accession number: NM_204725.2), and *Caspase-7* (GenBank accession number: XM_421764.7) coding sequences were cloned from DF-1 cells using the following primers: for chicken *GSDME*, 5′-ATGTTTGCAAAAGCAACAA-3′ (sense) and 5′-tcagaaaagctcctctac-3′ (antisense); for chicken *GSDMA*, 5′-atgtttaaaaaagtcacc-3′ (sense) and 5′-tcagtcggccctcgcagaac-3′ (antisense); for chicken *Caspase-3*, 5′-atgatgacagacataaaa-3′ (sense) and 5′-gcaaggaaagtagaattc-3′ (antisense); for chicken *Caspase-7*, 5′-atgtcaggagatcagcat-3′ (sense) and 5′-gaagtaaagttccttagt-3′ (antisense). pEGFP-N1-chGSDME, pEGFP-N1-chGSDME^NT^ (1–273 residues), pEGFP-N1-chGSDME^CT^ (273–505 residues), pEGFP-N1-chGSDME^NT^ (F2A), pRK5-Flag-chGSDME-HA, pET-21a-chCASP3, pET-21a-chCASP7, pGEX-6P-1-chGSDME, pRK5-Flag-chGSDME(D270A)-HA, pRK5-Flag-chGSDME(D273A)-HA, pRK5-Flag-chGSDME(AAAD)-HA, pRK5-Flag-chGSDME, pRK5-Flag-chGSDMA, pRK5-Flag-chGSDMA(DAVD), and pRK5-Flag-chGSDMA(FHPD) were constructed by standard molecular biology techniques.

### LDH assay

To determine cell death, DF-1 cells were seeded in 96-well plates and were incubated overnight, followed by different treatments. The LDH release from the cells was measured by cytotoxicity LDH assay kit (MCE, USA) per the manufacturer’s instructions.

### Flow cytometry analysis

To quantify pyroptotic cells with IBDV infection or Poly(I:C) stimulation, DF-1 cells were seeded on six-well plates and cultured overnight. Cells were infected with IBDV at an MOI of 1 or transfected with 1 µg/mL Poly(I:C). Twenty-four hours after IBDV infection or Poly(I:C) stimulation, cells were harvested and stained with anti-chGSDME^NT^ McAb for 50 min on ice, followed by Alexa Fluor 647 conjugated secondary antibody for 30 min on ice. After washes with ice-cold PBS, cells were stained with 10 µg/mL PI for 10 min at room temperature, and then analyzed with flow cytometry using CellQuest software (BD).

### Expression and purification of recombinant proteins

Chicken GSDME (chGSDME) was cloned into pGEX-6P-1 vector with N-terminal GST tag, and chicken caspase-3 and caspases-7 were cloned into pET-21a vector with C-terminal His tag. All plasmids were transformed into *E. coli* Rosetta (DE3) cells, and the transformants were grown in LB medium supplemented with appropriate antibiotics at 37°C. When the OD_600_ of culture medium reached 0.8, recombinant GST-chGSDME and His-chCASP7 expressions were induced with 0.4 mM IPTG at 18°C overnight. Recombinant His-chCASP3 expression was induced with 0.2 mM IPTG at 30°C for 6 h. The cultured cells were harvested and lysed by sonication in lysis buffer containing 50 mM Tris-HCl (pH 8.0) and 100 mM NaCl; the lysates were centrifuged at 12,000 rpm at 4°C for 30 min, and the supernatants were collected and filtered through a 0.45 µm syringe filter (Millipore, USA). Recombinants GST-chGSDME and His-chCASP3/7 were purified using glutathione sepharose (GE Healthcare, USA) and Ni-NTA agarose (Qiagen, Germany), respectively, per the manufacturer’s instructions. The GST tag was removed by overnight PreScission protease digestion at 4°C. chGSDME was further concentrated using an Amicon Ultra centrifugal filter with 10 kDa cut-off (Millipore, USA) and the buffer changed to PBS (pH 7.2).

### *In vitro* chGSDME cleavage assay by SDS-PAGE

To assay *in vitro* cleavage of chGSDME by caspases, 5 µg of purified chGSDME was incubated with different units of chCASP3/7 in a 20-µL reaction buffer (containing 10 mM PIPES (pH7.2), 100 mM NaCl, 10% sucrose, 0.1% CHAPS, 1 mM EDTA, and 10 mM DTT) at 37°C. At 60 min after incubation, 4 µL of 6 × SDS loading buffer was added to the reaction mixture and heated to 95°C for 10 min, and the cleavage of chGSDME was examined by Coomassie blue staining of the reaction samples separated on the SDS–PAGE gel.

### Confocal microscopy

To observe the subcellular localization of different domains of chGSDME, the DF-1 cells were seeded on 35 mm cover glass-bottom culture dishes and incubated overnight, followed by transfection with plasmids encoding different chGSDME mutants. At 20 hours after transfection, cells were observed with a laser confocal scanning microscope (Nikon C1 standard detector, Japan).

To observe the dsRNA in DF-1 cells infected with IBDV, the DF-1 cells were seeded on 35 mm cover glass-bottom culture dishes and incubated overnight, followed by infected with IBDV at an MOI of 1. At 16 h after infection, cell cultures were fixed with 4% paraformaldehyde, permeabilized with 0.1% Triton X-100, blocked with 1% bovine serum albumin (BSA), and then incubated with anti-dsRNA antibodies followed by TRITC-conjugated goat anti-mouse IgG antibodies. Nuclei were counterstained with DAPI (4′,6-diamidino-2-phenylindole). The cell samples were observed with a laser confocal scanning microscope (Nikon C1 standard detector).

### Western blot assay

Cell lysates were mixed with 6 × SDS loading buffer and boiled for 10  min. The samples were subjected to SDS-PAGE and then transferred to polyvinylidene difluoride (PVDF) membranes. After blocking with 5% skimmed milk, the membranes were probed with a primary antibody, followed by a HRP-conjugated secondary antibody. Finally, protein bands were visualized using enhanced ECL kit.

To examine HMGB1 release, cell culture supernatants were precipitated by methanol/chloroform method (78). Briefly, cell culture media were centrifuged at 5,000 rpm for 3 min to remove cell debris. The supernatants were precipitated by the addition of an equal volume of methanol and 0.25 volumes of chloroform, followed by centrifugation at 12,000 rpm for 10 min. The upper layer was discarded, and the same volume of methanol was added to the sample, followed by centrifugation at 15,000 rpm for 10 min. The supernatants were discarded, and the pellets were dried at 50°C, followed by resuspension with 1 × SDS loading buffer and boiled for 10 min. The samples were analyzed with Western blot as described above.

### Purification of IBDV genomic dsRNA

The IBDV genomic dsRNA was purified as described previously (79). DF-1 cells were infected with IBDV at an MOI of 0.01. Forty-eight hours post IBDV infection, cell culture supernatants were collected and centrifuged at 8000 rpm at 4°C for 3 min to remove cell debris. Then the supernatants were purified and concentrated using the Virus Concentration Kit (Beyotime, China). The concentrated virus particles were treated with 1% SDS and 1 mg/mL proteinase K at 56°C for 30 min to remove viral protein and release IBDV genomic dsRNA. Then the dsRNA was extracted using Viral RNA Rapid Extraction Kit (Aidlab Biotechnologies, China) per the manufacturer’s instructions.

### RNA interference

To knock down the expression of chGSDME in DF-1 cells, the siRNAs were designed and synthesized by Genepharma Company (China). The sense sequences of siRNAs for targeting chGSDME in DF-1 cells were 5′-GCAGAAGCCCAAGUAUCAUTT-3′ (RNAi#1), 5′-GCACUACGAAGAUAGUAAATT-3′ (RNAi#2), and 5′-GCUAUUGUUGCAGAACUAUTT-3′ (RNAi#3). DF-1 cells were seeded on 12-well plates and cultured overnight, followed by transfection with the siRNAs using RNAiMAX reagent. Double transfections were performed at a 24-h interval. Twenty-four hours after the second transfection, the expression of chGSDME in cells were examined.

### Generation of chGSDME- or MDA5- deficient DF-1 cells by CRISPR-Cas9 technique

The target sequence used for *chGSDME* is GCTTGTCTGAGGCGTTCAAG, and the target sequence used for *chMDA5* is CAGTGAACCATCCGGGGTCG. The sgRNAs sequences were cloned into lentiCRISPR v2 plasmid (Addgene: #52961). DF-1 cells were transfected with lentiCRISPR v2-sgchGSDME/sgchMDA5. Two days after transfection, the sgRNA-expressing cells were selected with puromycin, followed by the limiting dilution assay. The single clones were cultured in 96-well plates for 10–14 days. *chGSDME* or *chMDA5*-deficient clones were screened with Western blot and further verified by DNA sequencing.

### Measurement of IBDV growth in DF-1 cells

WT or chGSDME-KO DF-1 cells were infected with IBDV at an MOI of 0.5, and the whole cell cultures or the supernatant of cell cultures were collected at different time points (12, 24, 36, and 48 h) after infection. The viral titers of the collected samples were detected using TCID_50_ assay as described previously (28).

### Statistical analysis

The statistical analysis was performed using GraphPad Prism 8.0. The significance of the differences was determined by Student’s *t* test. The data are presented as means ± standard deviation, ***, *P* < 0.001; **, *P* < 0.01; *, *P* < 0.05; ns, *P* > 0.05.

## Data Availability

The data that support the findings of this study are available on request from the corresponding author.

## SUPPLEMENTAL MATERIAL

Supplemental figures jvi.01588-24-s0001.doc Figures S1 to S5.

ASM does not own the copyrights to Supplemental Material that may be linked to, or accessed through, an article. The authors have granted ASM a non-exclusive, world-wide license to publish the Supplemental Material files. Please contact the corresponding author directly for reuse.
