# Supplementary material for: Chicken GSDME, a major pore-forming molecule responsible for RNA virus-induced pyroptosis in chicken
Source: J Virol. 2024 Nov 22;99(1):e01588-24. doi: 10.1128/jvi.01588-24 (PMC11784259; doi:10.1128/jvi.01588-24)
Supplement: Supplemental figures — Figures S1 to S5. [file jvi.01588-24-s0001.doc]

**Supplemental figures**


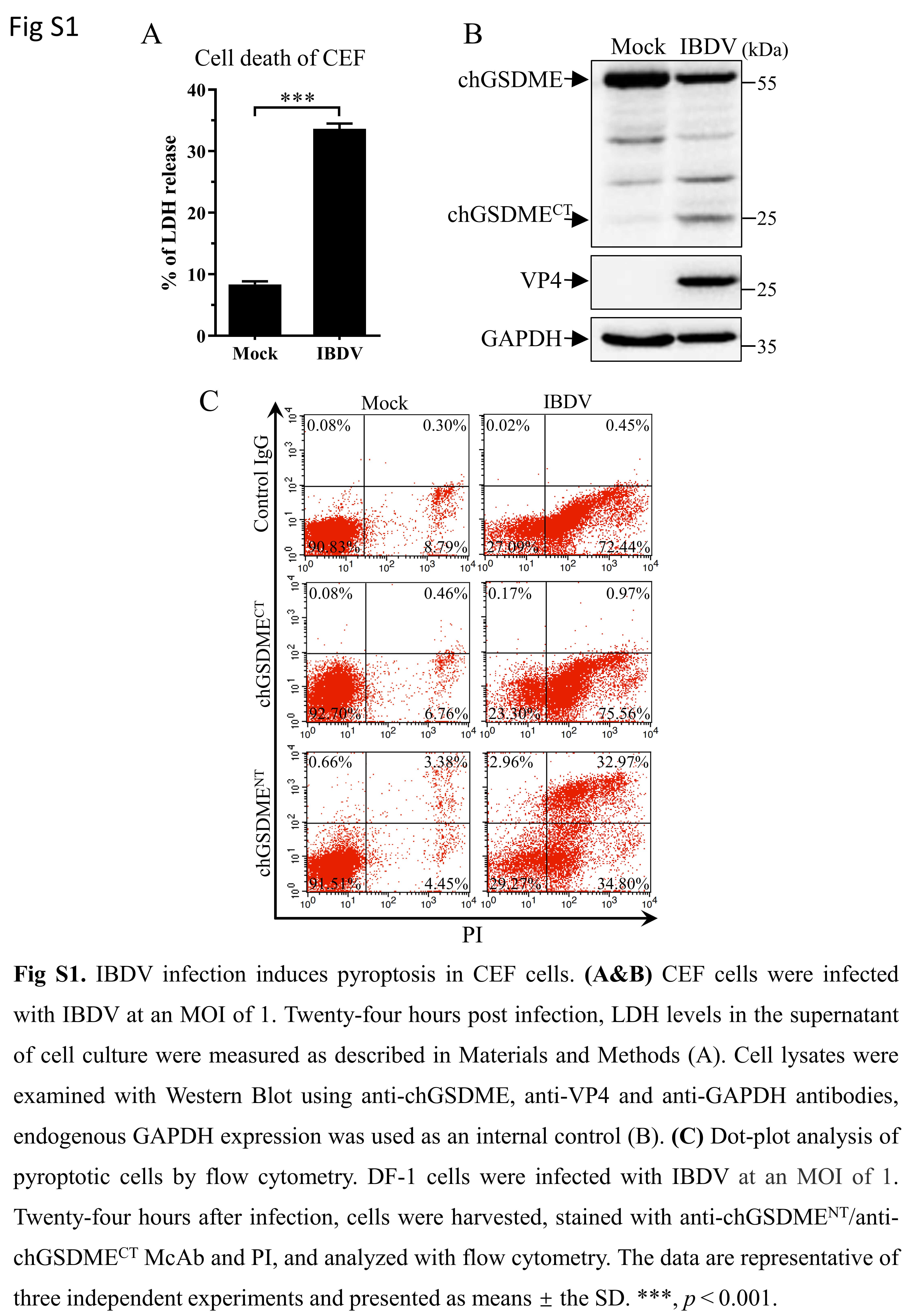


**Fig. S1.** **IBDV infection induces pyroptosis in CEF cells.** **(A&B)** CEF cells were infected with IBDV at an MOI of 1. Twenty-four hours post infection, LDH levels in the supernatant of cell culture were measured as described in Materials and Methods (A). Cell lysates were examined with Western Blot using anti-chGSDME, anti-VP4 and anti-GAPDH antibodies, endogenous GAPDH expression was used as an internal control (B). **(C)** Dot-plot analysis of pyroptotic cells by flow cytometry. DF-1 cells were infected with IBDV at an MOI of 1. Twenty-four hours after infection, cells were harvested, stained with anti-chGSDME^NT^/anti-chGSDME^CT^ McAb and PI, and analyzed with flow cytometry. The data are representative of three independent experiments and presented as means ± the SD. ***, *p* < 0.001.


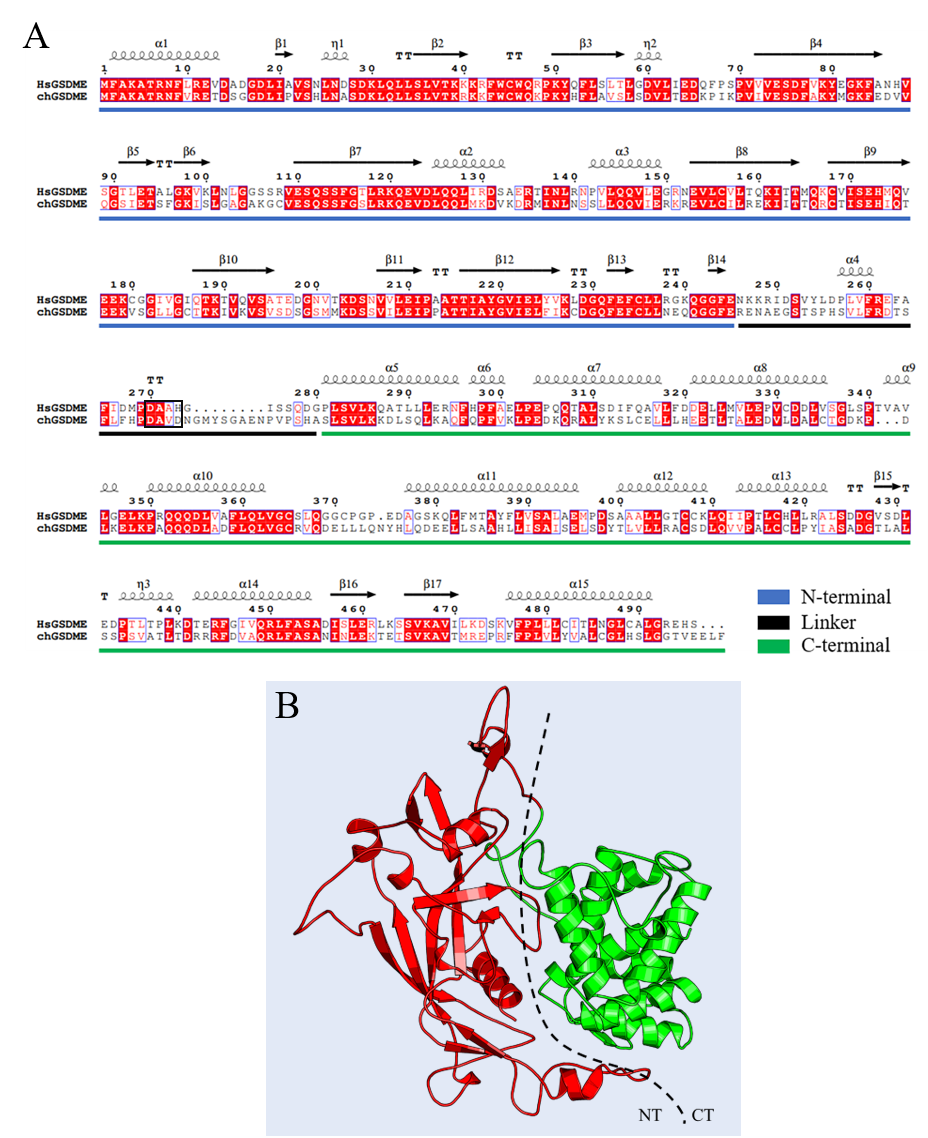


**Fig. S2.** **chGSDME shares a relatively high level of structural similarity with HsGSDME.** **(A)** Sequence alignment of chGSDME with HsGSDME. The alignment was generated using the ClustalW2 algorithm and presented using ESPript 3.0 (http://espript.ibcp.fr/ESPript/cgi-bin/ESPript.cgi). Identical residues are indicated in dark red, and conserved residues in red. The CASP cleavage tetrapeptide motif of chGSDME are indicated by black line in rectangular. **(B)** The overall three-dimensional structure model of chGSDME was generated by SWISS-MODEL (https://swissmodel.expasy.org/). The chGSDME-N (red) and -C (green) domains were separated by a dotted line.


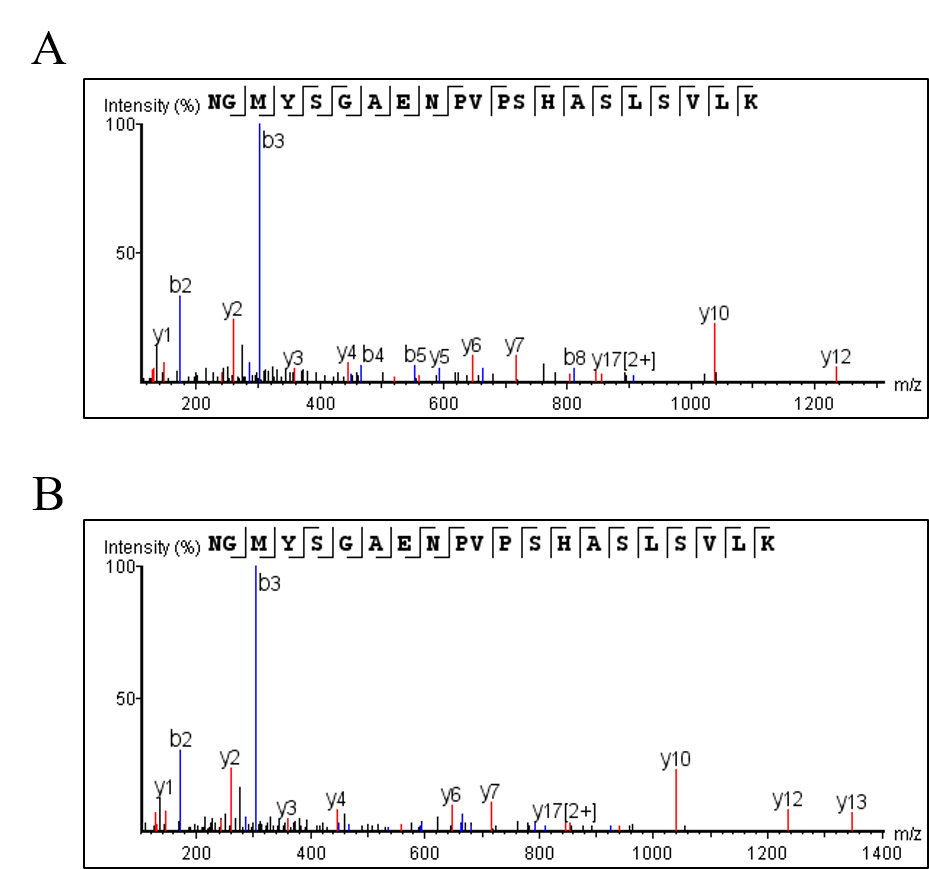


**Fig. S3**. **The results of mass spectrometry analysis for the N-terminal sequence of the cleavage products. (A)** N-terminal sequencing of ~25kDa cleavage product of chGSDME by chCASP3. **(B)** N-terminal sequencing of ~25kDa cleavage product of chGSDME by chCASP7.


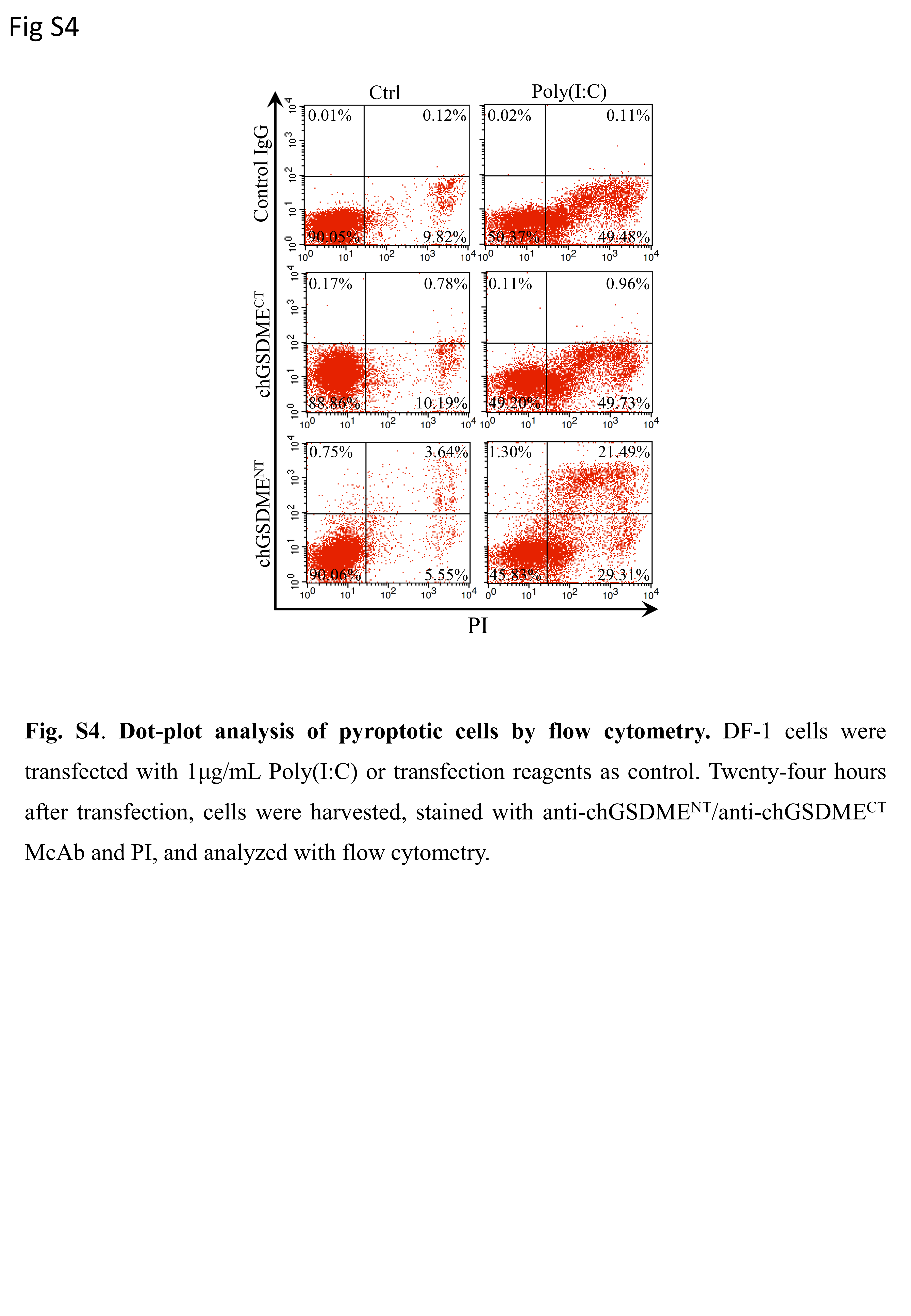


**Fig. S4. Dot-plot analysis of pyroptotic cells by flow cytometry.** DF-1 cells were transfected with 1μg/mL Poly(I:C) or transfection reagents as control. Twenty-four hours after transfection, cells were harvested, stained with anti-chGSDMENT/anti-chGSDMECT McAb and PI, and analyzed with flow cytometry.


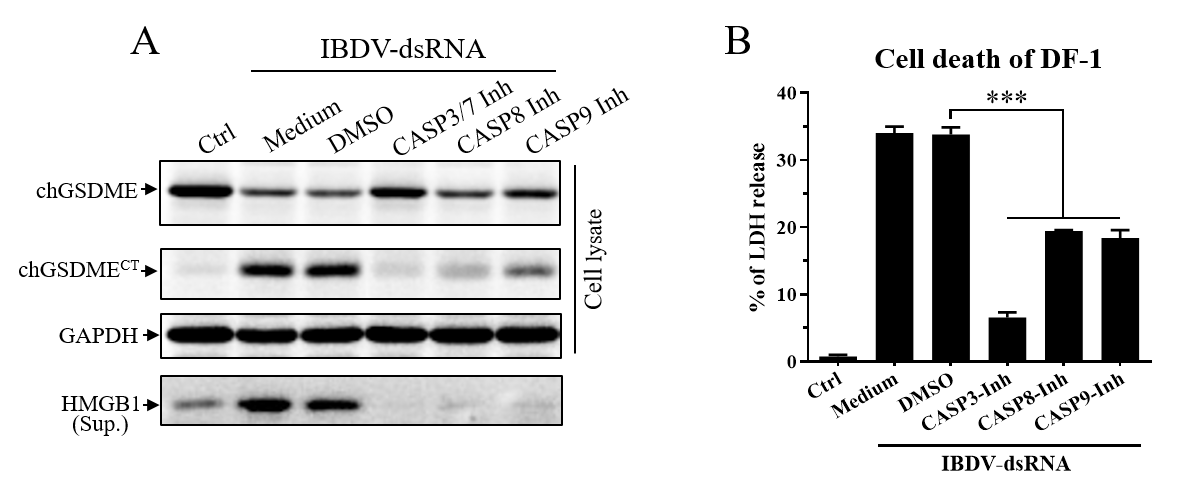


**Fig. S5. Caspase inhibitors restrict pyroptosis induced by IBDV-dsRNA.** DF-1 cells were transfected with 1μg/mL IBDV-dsRNA or transfection reagents as control, followed by incubation with either 20μM Z-DEVD-FMK, 20μM Z-IETD-FMK, 20μM Z-LEHD-FMK or DMSO as control. Twenty-four hours post IBDV-dsRNA stimulation, cell lysates and cell culture supernatants were examined with Western Blot using anti-chGSDME, anti-HMGB1 and anti-GAPDH antibodies, endogenous GAPDH expression was used as an internal control **(A)**. LDH levels in the supernatant of cell culture were measured as described in Materials and Methods **(B)**. The data are representative of three independent experiments and presented as means ± the SD. ***, *p* < 0.001.
